# Participation of children with disabilities in school: A realist systematic review of psychosocial and environmental factors

**DOI:** 10.1371/journal.pone.0210511

**Published:** 2019-01-29

**Authors:** Donald Maciver, Marion Rutherford, Stella Arakelyan, Jessica M. Kramer, Janet Richmond, Liliya Todorova, Dulce Romero-Ayuso, Hiromi Nakamura-Thomas, Marjon ten Velden, Ian Finlayson, Anne O’Hare, Kirsty Forsyth

**Affiliations:** 1 Occupational Therapy and Arts Therapies Subject Area, School of Health Sciences, Queen Margaret University, Edinburgh, Scotland, United Kingdom; 2 Department of Occupational Therapy & PhD Program in Rehabilitation Sciences, Boston University, Boston, United States of America; 3 School of Medical and Health Sciences, Edith Cowan University, Joondalup, Australia; 4 Occupational Therapy, Faculty of Public Health and Health Care, University of Ruse, Ruse, Bulgaria; 5 Department of Physical Therapy, Occupational Therapy Division, Faculty of Health Sciences, University of Granada, Granada, Spain; 6 Saitama Prefectural University, Graduate School of Health, Medicine and Welfare, Saitama, Japan; 7 Amsterdam University of Applied Sciences, Faculty of Health, School of Occupational Therapy, Amsterdam, the Netherlands; 8 Child Life and Health, SMC Research Centre, Edinburgh University, Edinburgh, Scotland, United Kingdom; Cincinnati Children’s Hospital, UNITED STATES

## Abstract

**Background:**

In order to make informed decisions about how best to support children and young people with disabilities, effective strategies that facilitate active and meaningful participation in school are required. Clinical factors, diagnosis or impairments somewhat helpful in determining what should be provided in interventions. However, clinical factors alone will not offer a clear view of how to support participation. It is helpful then to look at wider psychosocial and environmental factors. The aim of this review was to synthesise evidence of psychosocial and environmental factors associated with school participation of 4–12 year old children with disabilities to inform the development of participation-fostering interventions.

**Methods:**

A systematic search and synthesis using realist methods was conducted of published research. Papers had to include consideration of psychosocial and/or environment factors for school participation of children with disabilities. The review was completed in accordance with the Realist and Meta-narrative Evidence Syntheses: Evolving Standards (RAMESES) and Preferred Reporting Items for Systematic Review and Meta-Analysis (PRISMA) guidelines. Papers were identified via Boolean search of the electronic databases MEDLINE, CINAHL, PhycINFO and ERIC (January 2006-October 2018). Appraisal focussed on contributions in terms of whether the articles are appropriate for the review (relevance) and research quality (rigour). Data were analyzed using content and thematic analysis methods using a realist framework. A narrative synthesis of results was reported.

**Results and implications:**

We identified 1828 papers in the initial search. Seventy two papers were included in the final synthesis. Synthesis of findings led to three overarching mechanisms representing psychosocial factors for children (1) identity (2) competence and (3) experience of mind and body. Environmental aspects (context) compromised five interrelated areas: (1) structures and organization, (2) peers, (3) adults, (4) space and (5) objects. Our synthesis provides insights on how professionals may organize efforts to improve children’s participation. Consideration of these findings will help to proactively deal with suboptimal participation outcomes. Development of theoretically determined assessments and interventions for management of school participation are now required.

## Introduction

According to the World Health Organization’s World Report on Disability, an estimated 95 million children (5.1%) aged 0–14 years have some form of disability [1]. Common issues include Autism Spectrum Conditions, Developmental Delay, Behavioral Disorders and Learning Difficulties [1]. In many countries, these children have rights to be included in mainstream school [2–5]. For professionals who work with children this shift towards social and educational inclusion has meant that practices have had to evolve in tandem. Rehabilitation professionals now deliver a wide range of approaches to support early intervention and prevention for children with diverse needs. This includes school-based approaches alongside teachers and families to enable children’s full and active participation in school [6].

Participation or “involvement in life situations” [1] is a key outcome. Participation may take place anywhere. In this paper, we focus on the context of school. Participation in school includes unstructured activities (e.g friendships, play), organized activities (e.g. sports, clubs, arts), classroom based activities (e.g. group work, study) and engagement in social roles [7]. Children with disabilities are at significant risk for limited participation in school [8]. Such restrictions have significant lifetime consequences for achievement, quality of life and wellbeing [9–12]. There are several issues. Attendance for children with disabilities is reduced compared to peers [13]. Students with disabilities participate less in structured and unstructured activities, and experience reduced interaction and playground participation [14]. Children with disabilities additionally show less engagement in the wider school world, including clubs and organizations [13, 15].

Whilst there is an urgent need to develop interventions that promote participation in school, there is limited understanding of processes that may enable it [16]. Research to date has recognized the importance of psychosocial factors, though conclusions have been hampered by heterogeneous populations and variability in design and outcome measures [17]. There is little in the way of specific school based research to guide practices. Moreover, a requirement remains for comprehensive theories/models, as research has primarily considered individual psychosocial factors in isolation. A trend is departure from “medical” and “social” models. Both positions have been challenged as limiting [18]. The World Health Organization’s International Classification of Functioning, Disability and Health (ICF) [1] has been foundational to discussion with its definition of participation as “involvement in a life situation” and its assertion that the environment is a key determinant of participation (an integration of the “medical” and “social” models). However, the ICF has also provoked confusion and inconsistency in the field [16, 19, 20]. Everything people do is “involvement in a life situation” and participation is classified together with “activities” giving rise to conflicting interpretations [19, 21]. Driven by the ICF, there has also been a tendency to focus on a portfolio of actions done in everyday life [19]. Such indicators of “doing” say little about psychosocial drivers of participation such as motivation, social connection, preferences, choice and meaningfulness [19, 22, 23]. This paucity of theory leads to a situation whereby enhancement of participation outcomes is often an aspiration, but reliable, environmental or psychosocial interventions are not available.

To date only Imms et al. 2016 has conducted research which integrates various factors in a useful new direction [24]. Their narrative systematic review, although it did not focus on school specifically, concluded that the participation phenomenon is essentially dichotomous—requiring children to “attend” (be present) and also to be “involved” (engage, experience and so forth) [24]. A further insight has been to differentiate between participation and other influencing or “participation related constructs” which include preferences, sense of self and activity competence [24]. This work highlights the importance of careful definition, as well as identification of some import psychosocial factors. However, this work did not consider environment factors in detail, and was based on an analysis of randomized controlled trials (RCT) and intervention type studies only. Such restrictive inclusion criteria will have contributed to limiting the data that could have been available. RCTs rarely focus on context, detail on mechanisms of action or conceptual underpinnings [25]. Analysis of RCTs is less useful for answering conceptual or theory based questions [26]. Therefore, the present review employed a realist review approach to identify a broad range of environmental and psychosocial factors associated with participation, and to uncover the association between context, mechanisms and participation outcomes in school-aged children with disabilities to guide the development and implementation of interventions and assessments.

## Materials and methods

The United Kingdom Medical Research Council’s (MRC) guidance on development of complex interventions argues that new interventions must be underpinned by a conceptual framework and a theoretical understanding of the key processes underpinning an intervention [27]. This study uses realist review to address the requirement for theory and conceptual framework development outlined by the MRC. The process drew on systematic review and realist review methods. For systematic searching of the literature, we followed the PRISMA guidelines [28], as far as was relevant for a realist review. Realist methods were completed in accordance with the Realist and Meta-narrative Evidence Syntheses: Evolving Standards (RAMESES) guidelines [26].

We selected realist review as it meets requirements for dealing with complexity of both topic and research methods [25, 29, 30]. Realist review is an interpretive, theory-driven narrative summary which applies realist philosophy of exploring context, mechanisms and outcomes [25]. Developed in response to the weaknesses of traditional systematic review, realist review focusses on refining and developing theory. Realist reviews are organized around Context-Mechanism-Outcome (CMO) configuration [25]. Review aims to identify what works for whom, in what circumstances, in what respects and how, by identifying processes (mechanisms) that lead to outcomes in context [25, 29, 30]. The identification of open, embedded, interactive systems is central to the process of analysis. These assumptions constitute a realist philosophical ‘lens’ [25]. The steps of realist review are: (1) identifying the review question; (2) formulating the initial theory; (3) searching for primary studies; (4) selecting the studies and appraise their quality; (5) extracting, analyzing and synthesizing data. The details are described below [25].

### Identifying the question

The review question was: “What are the mechanisms and contexts which determine successful participation in 4–12 year old children with disabilities in school?” In developing the question, we drew from a range of perspectives. As the findings were indented for use internationally, the research team included professionals from several countries (Japan, the United Kingdom, Spain, Australia, Bulgaria, the Netherlands, and the United States of America). We ensured that team members represented a range of voices from those with an interest in participation in schools, representing expertise in psychology, rehabilitation, medicine, community pediatrics, neurodisability, community health sciences, education, occupational therapy, disability theory, and global health. The research team included professors, post-doctoral fellows and a range of senior academics and expert clinicians. A wider advisory group included rehabilitation, social care and educational practitioners and managers who provided regular input. Initial questions and review direction were discussed over email between the research team and advisory group. This included a discussion on realist informed approaches including an explanation of Context, Mechanism and Outcomes and the basics of realist theory. The research and advisory groups felt that the focus and question set was an authentic question which reflected curiosity about how schools were working and interest in understanding how to improve children’s participation.

### Formulating the initial theory

In line with a realist review approach, our initial thinking was informed by factors identified in the literature as possible key drivers of participation outcomes in school. Key literature was identified and synthesized through a scoping search [1, 10, 16, 17, 19–21, 23, 24, 31–37]. This initial scoping helped to identify theoretical areas, concepts and perspectives (a summary of the initial literature review is presented in Appendix A in S1 File). Amongst the main ideas considered were Bronfenbrenner’s ecological systems model [31], the World Health Organization’s International Classification of Functioning, Disability and Health (ICF) [1] and practice models to support participation [34]. Using the above scoping review, discussion and analysis amongst the research team and advisory group led to the development of initial mechanisms, contexts and outcomes, and the target population. Initial mechanisms focused on personal psychosocial factors which may drive participation outcomes: (1) children’s choices, initiative, interests and skills and (2) patterning and performance of participation, including routines (e.g. going to school, eating lunch, playing with friends) and roles. Initial thinking also considered psychosocial factors related to common issues experienced by children with disabilities including pain, anxiety, stress, or fatigue. In considering the context, we drew on ecological systems theory, focusing on the “microsystem” as the system closest to the person and the one in which they have direct contact [31]. In this case, the characteristics of classrooms and schools, denoting circumstances within school that may be considered as enablers or barriers. This approach meant that issues pertaining to context outside the school (for example, the role of parents, home life, or government policies) were not considered.

Participation in school was the outcome of interest. The most common definition of participation originates in the World Health Organization’s International Classification of Functioning Disability and Health referring to a person’s “involvement in life situations” [1]. As noted, other authors have criticized this definition [17, 38]. The definition used in the review builds on the ICF definition, but also implies that participation must be meaningful, with personal or social significance. Our definition reflects recent ideas [24] that participation has two essential components: attendance and involvement. The definition is presented in Table 1.

**Table 1:** Participation definition.

School participation includes active and meaningful (from a personal or socio-economic perspective) activities which are required or desired to fulfil the role of the school pupil within or around the school context. Participation in school is not only classroom activity, school work or achievement. Participation includes school events, trips, teams, clubs, relationships with adults and friendships with peers. School participation can be understood in terms of how much, how often and what activities the child does (attendance), as well as their subjective experience (involvement).

The target population was defined as children who have a physical, developmental, behavioral, or emotional condition and who also require health and education services of a type or amount beyond that required by children generally [39]. Middle child hood (4–12 years) was selected as the target group. During middle childhood (defined as ages 4 to 12), a child’s mastery of developmental challenges is strongly influenced by school experiences, hence exploring participation in this context is important. Children are moving from nursery/kindergarten provision to increasingly formal education settings, but have not yet entered the adolescent phase where a number of other unique challenges appear.

### Systematic searching process

Searches were conducted for English Language papers in MEDLINE, CINAHL, PhycINFO and ERIC databases covering the period January 2006 and November 2018. This span was chosen in order to capture a contemporary conceptualization of participation. Searching was completed by DM and SA. The search strategy utilized text word searching in the title or abstract along with database Subject Headings. Terms included disability “special needs”, “additional needs”, “disabled persons”, “motor disorders”, “developmental disabilities”, “intellectual disability”, “communication disorders”; age “child”, “children,” “pediatric”, “girl”, “boy”, “schoolchild”, “participation”, “inclusion,” “involvement”, “engagement”, “life situations,” “environment”, “surroundings”,”setting”, “context”, “school”, “education“, “class”, and “teacher”. Strategies were developed with support from an information professional (Appendix B in S1 File illustrates the strategy used in MEDLINE). In addition, a hand search compiled by DM and SA checked reference lists from relevant articles, including all those included in the review.

### Selection and appraisal of studies

Members of the research team screened a portion of the titles and abstracts (DM, SA, MR). The potentially relevant records identified by individual members of the research team were then discussed with the other authors to confirm eligibility. This was followed by screening the full text of potentially relevant studies to determine eligibility for inclusion.

Overall, if papers contained evidence relevant to school participation (or related proxy outcome, e.g. school attendance), for children with a disability/special educational need, with discussion of environmental and/or psychosocial factors, the document was retained for further review. In keeping with realist methods, selection criteria regarding study design were not predominant [25, 29, 30]. Methodologically, papers could include any type of peer-reviewed paper including intervention studies, observational research, qualitative research and literature reviews. Literature reviews may be included in realist review if they provide relevant theoretical insights [25, 29, 30]. We did reject all purely descriptive accounts (e.g. opinion pieces or editorials) and grey literature as there was ample peer-reviewed material. We also excluded psychometrics focused papers, due to their general interest in identifying what participation was, rather than its influencing factors. Children with disabilities were identified via medical diagnosis or other support needs (e.g. identified as requiring “special” education). Papers focusing solely on community or leisure participation were rejected, although papers which discussed school participation amongst other settings were included. We aimed to identify studies of relevance to middle childhood which we defined as 4–12 years. Studies close to this age range were passed onto the next stage for further assessment if the findings were viewed by the team as potentially relevant and generalizable to middle childhood. In some cases the assessment of age was not necessary, as the participants were teachers, parents or health professionals, and in the case of some reviews. Initial screening criteria are in Table 2.

**Table 2:** Initial selection criteria (titles and abstracts).

Inclusion criteria	Exclusion criteria
**School participation**	
Focus on school participation and psychosocial/environmental factors	Community, home or leisure participation only
**Publication type**	
Any type of primary research or literature review	Books, editorials, conference proceedings, commentaries, abstracts, theses, dissertations and other grey literature
**Publication period**	
January 2006 -October 2018	Prior to December 31^st^ 2005
**Publication language**	
Articles published in English	Published in languages other than English
**Population**	
Middle childhood (approx. 4–12 years old) with disability and/or other special/educational/health need.	Population is typically developing

In accordance RAMESES guidelines [26], final selection of papers focused on contributions in terms of whether articles were appropriate for the research question (relevance) and quality of evidence (rigour) [25, 29, 30]. This was an iterative process, and disagreements were dealt with via discussion (DM, SA and MR). Review of relevance was used to ensure a systematic process and to reduce selection bias. A system of questions was used to identify whether an article was relevant by examining content, insights provided by the study and focus (see Table 3). Assessment of rigour was used to judge quality, credibility and trustworthiness of evidence [25]. Each reviewer appraised each paper by asking key questions on research quality [40]. Papers could be excluded on the basis of relevance or rigour. Each paper was scored 0 (failed to meet criteria) or 1 (met criteria). Studies scoring 0 on either criteria were excluded.

**Table 3:** Rigour and relevance assessment.

Is the paper relevant enough? (relevance)
Do the questions/aims refer to participation of children with disabilities in the school context? If not, do they focus on related concepts (e.g. engagement, friendships,school work, activities, or roles?) and are the findings relevant to the review? If the sample mean does not include children aged 4–12 are the findings generalizable/transferable to the 4–12 age range? Does the study provide any insights about how children’s participation can be supported in school through interventions? Does the study provide insights about which factors (child or environment) are most important for school participation and why?
Is the paper good enough? (rigour)
Is the design appropriate? Is the context or setting adequately described? Is the sample adequate to explore the range of subjects and settings, and has it been drawn from an appropriate population? Was the data collection or review method adequately described and rigorously conducted? Was there evidence that the data analysis was rigorously conducted? Do any claims to generalisability follow logically, theoretically and statistically from the data?

### Data extraction, analysis and synthesis

Data were extracted using predefined forms by DM, SA and MR, regularly checking each other’s work. Data were extracted on: country and author; sample characteristics: sample size; participants’ age and gender; diagnostic category (if available); key findings; relevance and rigor mechanisms, contexts, and outcomes.

Analysis were done by DM, SA and MR following a staged process based on careful review, coding and frequent return to primary studies as necessary. Broad aspects of context and mechanism were identified and coded first. The key analytic process in realist review involves iterative testing and refinement of theoretically based explanations for why outcomes happen, using research papers as data sources [25, 29, 30]. In our case we were focused on participation in the school, and we attempted to find and synthesize evidence to demonstrate that particular mechanisms were important in generating school participation outcomes and to identify which aspects of context mattered. Data were synthesized using qualitative methods (content and thematic analysis) [41], using realist concepts as a framework [25, 26]. Context and mechanisms were operationalized using codes and sub-codes as in typical qualitative analysis [41]. In the early stages very many individual codes were created and grouped. For example all aspects relating to the child’s motivations were grouped into a broad “motivations” category and all aspects of the physical environment were grouped into a “physical environment” category. Specific aspects were then identified and coded with sub-codes, e.g. social aspects, physical access, or assistive devices. As analysis progressed, more refined codes were created and sorted and grouped to identify mutually exclusive categories of mechanisms and contexts which were coherent and could be designated a single unifying label.

As the analysis progressed, evidence of which mechanisms and context were important was carefully mapped against the emerging taxonomy. Tables were derived, including categories and sub-components, including each article relating to the sub-component. Regular meetings were held and interpretations shared across the research team and advisory group, including re-examination the original articles. Further refinement of the findings continued until agreement was reached. Following final assessment, two members of the research team reviewed once again the articles, and checked the findings. We also attempted to identify disconfirming data or data that might challenge or refute ideas. During this process there was a point at which no new categories of mechanisms or context emerged i.e. saturation was attained. Final labels were then assigned to each area and the narrative summary was written.

## Results

**Fig 1:**
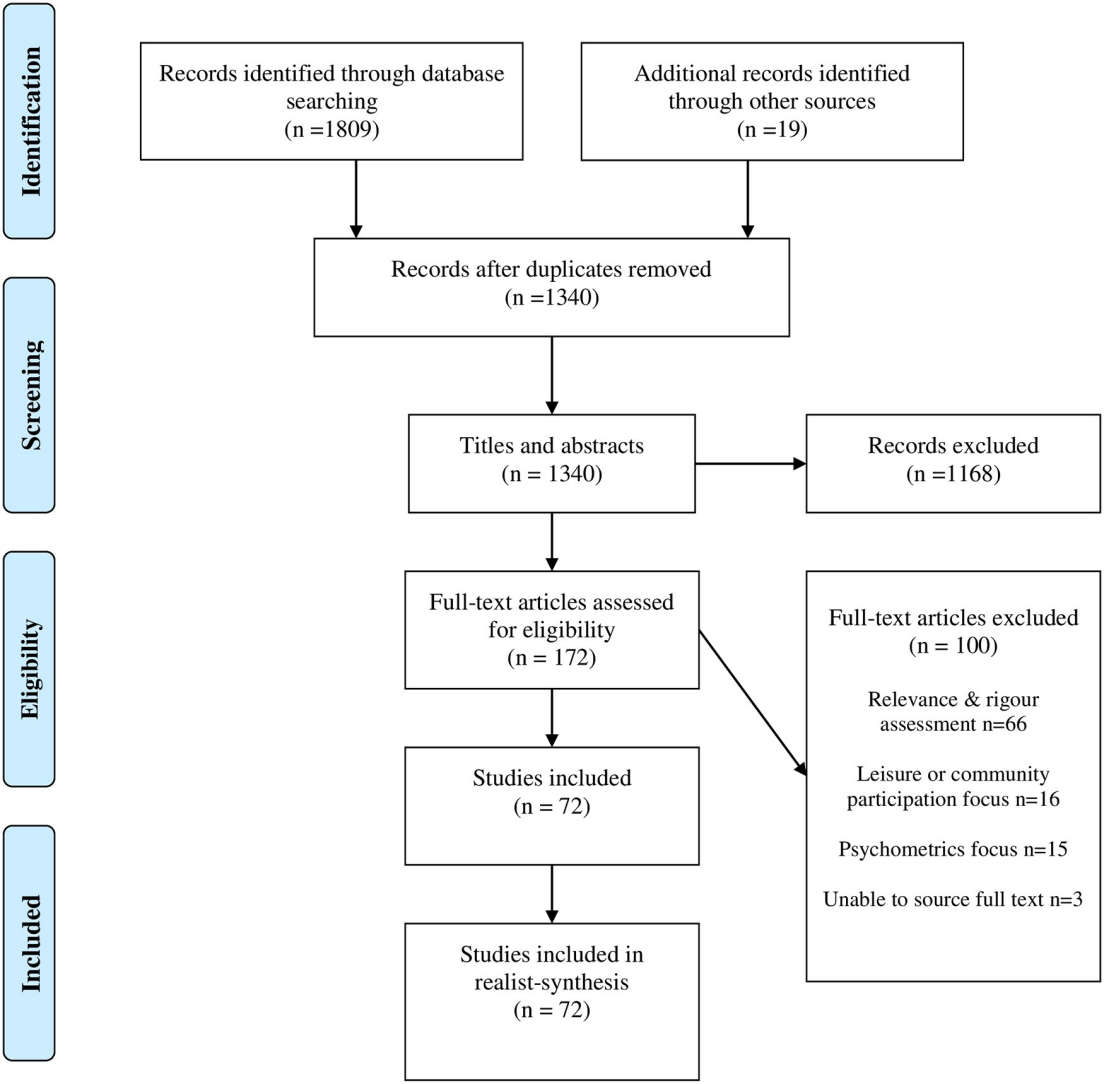
Study selection process (PRISMA diagram).

The electronic literature search and hand search identified 1828 papers, 1168 of which were removed at the title and abstract stage. Next, 172 papers were reviewed in full. On review, 100 papers were excluded, leading to 72 papers in the final synthesis (Fig 1) (full details of all papers are provided in Appendices C and D in S1 File). Type of disability was consistent with issues commonly seen in schools (including Autism Spectrum Disorders, Cerebral Palsy, Learning Disability, Learning Difficulty, Developmental Delay, and Physical Disabilities) (Appendices C and D in S1 File). Forty-six percent (n = 33) of the research was quantitative in design (including trials, cross sectional studies, observational studies and quasi-experimental studies), with the remainder consisting of mixed-methods (n = 4, 5%), qualitative (n = 17, 24%) and review papers (n = 18, 25%). Sample size ranged from 6 to 47 participants in qualitative research, and 14 to 3,752 participants in quantitative (excluding two very large national studies ranging from 18,119 to 64,076 (weighted) participants) (Appendix C in S1 File). Studies from Europe (n = 28), the USA and Canada (n = 22) accounted for 70% of papers with the remainder coming from Australia (n = 11), Brazil (n = 1), Chile (n = 1), Israel (n = 4), Japan (n = 1), New Zealand (n = 1), Taiwan (n = 1), India (n = 1) and Thailand (n = 1) (Appendices C and D in S1 File).

**Fig 2:**
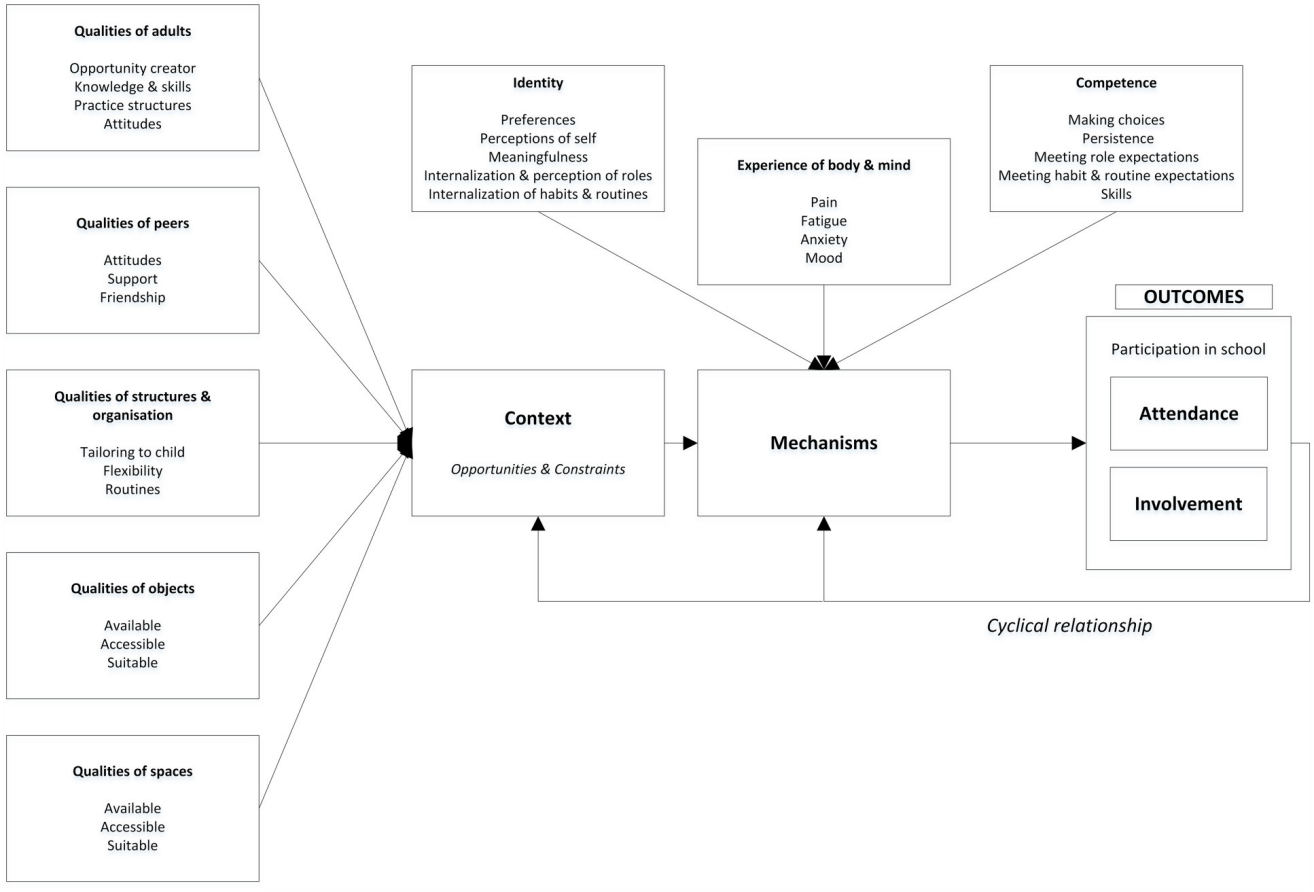
Conceptual framework of mechanisms, contexts and outcomes. Context and mechanisms hypothesized to be vital intervening factors in predicting children’s participation. Context provides opportunities and constraints. Mechanisms drive participation outcomes. Participation as an outcome has two components: attendance and involvement [24]. As children participate, they experience feelings, sensations and perceptions which may be adaptive or maladaptive (e.g. enjoyment, boredom, amusement). There is a cyclical relationship between participation, context and mechanisms.

The initial analysis identified 72 contexts and 79 mechanisms. These were the psychosocial child and environment factors driving participation outcomes in schools. Further analysis revealed three synthesized mechanisms, and five synthesized contexts. Based on the evidence, we constructed a conceptual framework that depicts mechanisms and contexts influencing school participation for children with disabilities (Fig 2). Details on specific categories of mechanisms and context are provided below.

### Mechanisms

Synthesis of findings led to three overarching mechanisms representing psychosocial issues and the child’s experiences (supporting studies are presented in Table 4 and Appendix E in S1 File).

(1) **Identity**—these mechanisms were associated with “being”, or the thoughts and feelings the child had about themselves (e.g. believing in themselves, having confidence, understanding their roles or feeling a like a member of the school community) as well as perceptions of activities and tasks in school (e.g. interests, preferences or perceived enjoyment).
(2) **Competence**—these mechanisms were associated with “doing” or what the child did in school (e.g. following rules, showing interest, being confident, or following a routine).
(3) **Experience of mind and body (symptoms)**–these mechanisms were associated with issues commonly experienced by children with disabilities in schools: pain, anxiety, mood and fatigue/tiredness.

**Table 4:** Mechanisms.

Category	Mechanisms	Mechanism aspects	Supporting evidence
Identity	**Preferences**	Interests; perceived enjoyment; attraction to activities	[14, 24, 42–46, 46–52]
	**Perceptions of self**	Self-esteem; self-efficacy; confidence; perceived competence	[24, 46, 48–58]
	**Meaningfulness**	Willingness; perceptions of satisfaction	[14, 21, 24, 45, 50, 51, 59, 60]
	**Internalization & perception of roles**	Understanding & knowledge of roles; feeling like a ‘legitimate’ participant; feeling included; feeling membership & school identity	[56, 57, 61]
	**Internalization of habits & routines**	Familiarity, knowledge, preparedness, and automaticity of habits and routines	[44, 53, 62–64]
Competence	**Making choices**	Showing initiative; being proactive; acting on interests	[14, 24, 44, 52, 53, 65–68]
	**Persistence**	Working towards goals; perseverance; independence; self-reliance; being committed	[45, 47, 51–53, 57, 68–72]
	**Meeting role expectations**	Following rules and norms; fulfilling role expectations; routine performance in school and other roles	[57, 61, 70, 71, 73]
	**Meeting habit & routine expectations**	Having routines; following routines; having habits; doing what’s expected	[55, 62]
	**Organisation & planning skills**	Sequencing; concentration; memory; organization skills	[35, 46, 49, 51, 52, 55, 56, 63, 70, 72, 74]
	**Motor skills**	Gross and fine motor skills	[8, 14, 49, 51, 52, 55, 58, 70–72, 74–80]
	**Communication skills**	Expressive/receptive language; social communication skills	[14, 15, 42, 49, 51–53, 67, 72, 74]
Experience of mind and body (symptoms)	**Pain**	Cognitions; catastrophizing; withdrawal	[8, 14, 52, 71, 75, 79, 81–83]
	**Fatigue**	Energy level; fluctuating symptoms; sleep disturbance; withdrawal	[14, 46, 58, 71, 80, 84–86]
	**Anxiety**	Fear; frustration; anger; aggression; withdrawal	[46, 51, 53, 58, 60, 71, 80, 87–89]
	**Mood**	Sadness; depression; withdrawal	[13, 52, 71, 80, 81, 84, 89]

#### Identity

Analysis indicated that these mechanisms related to the child “being”, and how children perceived and made sense of their participation within school. Firstly, the information extracted from studies overwhelmingly and specifically demonstrated the relevance of mechanisms related to motivations, preferences, and interests. The key mechanisms were children’s own interests and preferences including selection of certain activities based on interests/preferences and perceptions around potential enjoyment (or not) of activities which motivated choices [14, 24, 42–46, 46–52]. Participation was also strongly influenced by children’s self-perceptions, including self-esteem, self-efficacy, confidence, and perceived competence—all of which influenced children’s activity in-the-moment and over time, influencing current and future participation[24, 46, 48–58]. Perceptions around meaningfulness were also identified as an influencer of participation, including perceptions around activities that were valued or especially significant to children, as well as perceptions of personal satisfaction and pleasure associated with activities[14, 21, 24, 45, 50, 51, 59, 60].

Children’s internalization and understanding of routines and habits emerged as conditions influencing participation in school. Studies highlighted children’s internalization of habit/routine, familiarity with habit/routine and automaticity of habit/routine were mechanisms for participation [44, 53, 62–64]. Knowing the steps involved in activities or routines led to reduced demands on the child to understand, process, or plan, and when internalized as patterns of actions, facilitated participation by providing a set of rules to navigate the school context. Routines of the school day were noted to shape children’s daily participation, with references to the fact that children’s participation in school was supported by structured activities and programs [44, 64], and that regularized activities in the classroom supported participation for children with disabilities [44]. Children themselves perceived that rules, norms and routines are important in structuring their participation [62]. Parents also indicated that routines influence participation [44, 53].

Children’s knowledge, understanding and subjective experience of roles influenced their participation. In the school, possible roles included being a pupil, friend or member of a club. Disabled children tended to occupy less “desirable” roles within the school. Roles considered desirable by children, especially those including being good at something (e.g. best in class) or “best friend” roles were seldom held by children with disabilities [61]. Children with disabilities also engaged in less “doing roles” (such as athlete, leader, helper and tutor) and more were likely to be classified into negative roles including “challenged learner”, victim or bully [61]. Mechanisms influencing participation were internalization of roles (either positive or negative roles), leading to positive or negative self-perceptions, and understanding/knowledge of role requirements [56, 61]. Related mechanisms included self-perceptions relating to inclusion, focusing on children’s subjective experiences of social inclusion, sense of membership and sense “school” identity [57].

#### Competence

Competence mechanisms reflected “doing” or behavioral aspects and how children engaged in participation. Well-supported mechanisms enabling participation were children taking initiative, being proactive and acting on interests [14, 24, 44, 52, 53, 65–68] Research also demonstrated that seeking independence and autonomy, showing responsibility and commitment, displaying persistence and perseverance were drivers of participation [45, 47, 51–53, 57, 68–72].

Other competence mechanisms related to following routines and having daily habits [55, 62] as well as consistency of behavior, including being predictable, being systematic and preparedness for routines [55, 62]. Also identified as important conditions for participation were children meeting teachers’ expectations and following the school’s rules [61]. Finally, patterns of behaviors that followed from particular roles were identified as shaping quality and quantity of participation, including patterns of behaviors associated with friendship roles and patterns of behaviors associated with school-based roles (for example sports team member) [57, 61, 70, 71, 73].

Studies exploring relationships between skills and participation were common. In total, 27 papers provided data. However, researchers are now concluding that deficits or improvements in skills, although related to participation, are not related in a direct or linear fashion. The evidence challenges the idea that an increase in skill equates to an increase in participation. Psychological characteristics, personality and preferences are also important [14]. The evidence did indicate, however, that skills were important for the completion of certain types of activities in certain situations. For example, social skills are often required to access play situations [57]. The mechanisms related to skills identified as important for participation were organisation and planning (e.g. sequencing, concentration and memory) [35, 46, 49, 51, 52, 55, 56, 63, 70, 72, 74]; communication/social skills [14, 15, 42, 49, 51–53, 67, 72, 74] and motor skills [8, 14, 49, 51, 52, 55, 58, 70–72, 74–80].

#### Experience of mind and body

The literature provided good support for the influence of symptoms associated with disability on participation. These were pain, fatigue, anxiety and mood. Twenty-two papers provided data. Identified mechanisms were concerned with experiences related to symptoms. These were: pain (especially cognitions and catastrophizing) [8, 14, 52, 71, 75, 79, 81–83]; fatigue, including lowered energy, tiredness, and sleep disturbance[14, 46, 58, 71, 80, 84–86]; anxiety and its consequences including fear, frustration, and anger [46, 51, 53, 58, 60, 71, 80, 87–89], and low mood, sadness or depression [13, 52, 71, 80, 81, 84, 89].

Fundamental underpinnings were closely related across the different symptoms, drawing on social learning and cognitive-behavioural theory, suggesting that illness behaviours or responses generate negative behavioural patterns which may be maintained and strengthened over time [8, 13, 71, 75, 81, 83]. These mechanisms lead to reduced participation through disengagement from activity and a cyclical pattern of attempts to control symptoms through increasing withdrawal from activities.

### Contexts

The next step was to explore how and which contexts facilitated or provided opportunities for participation versus contexts which restricted/constrained participation. This twofold role of context was evident throughout. Context comprised five interrelated areas: (1) structures and organization of the school, (2) peers, (3) adults, (4) physical spaces and (5) objects. Sub-components of each area were identified by the reviewers, focusing on opportunities (supports) or constraints (barriers) to school participation (supporting studies are presented in Table 5 and Appendix F in S1 File).

**Table 5:** Contexts.

Context	Sub-component	Opportunities & Constraints	Supporting evidence
**Structure & organization**	**Tailoring to child**	Opportunity: Equal opportunities; responsive to needs; individualized; child mediated Constraint: Not individualized	[10, 15, 17, 21, 24, 35, 42–44, 49, 52, 54, 57, 58, 61–65, 67–70, 72, 80, 90–96]
	**Flexibility**	Opportunity: Adaptable; flexible Constraint: Rigid	[10, 14, 15, 20, 35, 36, 42, 44, 52, 53, 57, 58, 64–66, 68, 69, 71, 80, 91, 96–98]
	**Routines**	Opportunity: Consistent; predictable; planned; collaborative Constraint: Unpredictable; unstructured and/or lacking of rules or regulations	[10, 14, 44, 67–69, 72, 84, 92, 93, 96–98]
**Adults**	**Opportunity creator**	Opportunity: Provide opportunities for participation; shape positive roles Constraint: Shape negative roles	[16, 21, 42, 43, 49, 52, 57, 61, 64, 66, 96, 97, 99]
	**Attitudes**	Opportunity: Positive attitudes Constraint: Unsympathetic attitudes	[10, 14, 44, 49, 53, 56, 58, 71, 72, 80, 93, 96, 99, 100]
	**Knowledge & skills**	Opportunity: Competent; knowledgeable Constraint: Lacking in knowledge	[14, 35, 44, 49, 62, 96, 101]
	**Practice structures**	Opportunity: Collaboration between staff Constraint: Poor communication between staff	[14, 35, 69, 93, 96, 101, 102]
**Peers**	**Support**	Opportunity: Encouraging; practical and emotional support Constraint: Discouraging; bullying; discrimination	[10, 15, 16, 21, 36, 43–46, 49, 57, 58, 60, 61, 64, 68, 71, 73, 80, 92, 99, 100]
	**Friendship**	Opportunity: Nurturing relationships; opportunities for friendship Constraint: Friendship avoidance	[15, 43, 49, 60, 61, 66, 71, 73, 87]
	**Attitudes**	Opportunity: Positive attitudes Constraint: Negative attitudes; stigma	[10, 24, 36, 46, 53, 58, 69, 78, 80, 92, 93, 99, 100]
**Spaces**	**Available/ Accessible**	Opportunity: Spaces exist; spaces usable as required Constraint: Spaces inaccessible	[10, 14, 21, 24, 44, 45, 49, 52, 59, 60, 64, 65, 67, 69–71, 97, 100, 101]
	**Suitable**	Opportunity: Design “just right”‘; layout “just right”; sensory qualities attended and modifiable Constraint: Crowded; unfamiliar; sensory qualities unmodified/unsuitable	[10, 14, 35, 47, 52, 53, 60, 65, 68, 71, 78, 92, 96, 99, 103]
**Objects**	**Available/ Accessible**	Opportunity: Objects exist; objects are usable and acceptable Constraint: Objects are unavailable	[10, 14, 21, 24, 35, 36, 50, 52, 53, 59, 60, 62, 63, 65, 72, 86, 91, 95, 101]
	**Suitable**	Opportunity: Objects address needs Constraint: Objects are complicated; usability issues; cumbersome; unsuitable; isolating	[14, 35, 44, 50, 52, 53, 60, 71, 99]

#### Structure and organization

Structure and organization was a well-supported aspect focusing on the ways things were done in the school. Facilitative aspects were described as being tailored to the child, responsive to needs, individualized, and child led [10, 15, 17, 21, 24, 42–44, 49, 52, 54, 57, 58, 62–65, 67–70, 80, 90–94, 96]. Facilitative structures/organization were also described as adaptable and flexible [10, 14, 36, 44, 52, 64, 65, 68, 69, 80, 96, 97, 98], predictable [44] and well-planned [10, 68, 69, 93, 96, 98]. The most common constraint to participation identified was lack of individualization [15, 20, 42, 44, 49, 53, 57, 58, 65, 66, 69, 71, 72, 80, 91, 97, 98]. Other identified constraints included rigid routines [10, 14, 67, 80, 84, 92, 93, 97, 98] or routines which were unpredictable or disordered [70, 72].

#### Adults

There was abundant evidence that adults (referring to teachers and other staff within school) were key in creating opportunities for participation and were also influential in shaping the quality, frequency and range of children’s roles [16, 21, 42, 43, 52, 57, 61, 64, 66, 96, 97, 99]. Adult’s positive and sympathetic attitudes were facilitative of participation [14, 93, 96, 99, 100] as were individuals who were competent and knowledgeable [44, 62,49, 68, 96, 99]. Good collaboration between adults was also facilitative [14, 68, 93, 96, 99]. Attitudes were identified as restrictive, as well as adults who were unsympathetic [10, 44, 47, 53, 58, 71, 72, 80, 93, 99, 100] or lacking in knowledge [14, 35, 49, 101]and institutional collaboration [35, 69, 99]. Adults were also noted to play a part in shaping negative roles (e.g. by ‘pigeonholing’ children with disabilities as less able and therefore offering them fewer participation opportunities, or by being reluctant to allow students to learn or play independently) [61].

#### Peers

The evidence indicated that facilitative peers (referring to other children within the school) provided practical and emotional support enabling participation [10, 15, 16, 21, 43, 44, 46, 49, 57, 58, 60, 64,68]. Facilitative peers also provided opportunities for friendship [43, 49, 61, 71, 73]. Positive attitudes were also identified as important in creating opportunities for participation [24, 36, 46, 69, 93, 100]. Studies also identified non-supportive actions and behaviours, including bullying [15, 43–45, 57, 61, 71, 73, 80, 92, 99], negative attitudes [10, 46, 53, 58, 78, 80,92, 93, 100], and friendship avoidance [15, 49, 60, 66, 71, 87].

#### Spaces

Supportive spaces were described as being accessible and usable [10, 14, 21, 24, 36, 44, 45, 59, 60, 65, 67, 68, 69, 71, 78, 97, 99] with suitable design/layout and suitable sensory qualities [53, 60, 62, 68, 86, 96, 99]. Constraints to participation focused on restricted access to areas where activities happen [10, 14, 35, 47, 49, 52, 53, 60, 65, 71, 78, 92, 97, 99, 103]. Other issues included unsuitable sensory qualities, spaces which were unfamiliar, and spaces which were crowded or difficult to navigate [35, 52, 53, 60, 65, 97,99].

#### Objects

Research on objects focused on the availability of objects needed to participate in specific activities, for example, wheelchairs and assistive devices [10, 14, 21, 24, 35, 36, 52, 59, 60, 62, 63, 65, 91, 99]. Usability and acceptability to the child were noted as important [14, 44, 50, 52, 53, 60, 71, 72, 99]. Research on constraints associated with objects was fairly limited. Objects being unavailable [10, 60, 65, 72, 99], difficult to use [35, 53, 50, 99] or isolating/stigmatizing [45, 50] were identified as constraints to participation.

## Discussion

This realist review has developed a conceptual framework for children’s school participation, and identified the processes (mechanisms) and contexts influencing participation outcomes. The synthesis is of key issues that decision-makers and interventionists may consider to help children to participate in school.

The findings support the hypothesis that identified mechanisms and contexts are important factors associated with participation outcomes. Specifically, the findings show mechanisms in three clusters focusing on identity, competence and the child’s experience of mind and body. The context (environment) is conceptualized in terms of adults, peers, the schools’ structures and routines and spaces/objects. Unlike most models designed for dealing with specific impairments or diagnoses, this model is useful with any child with any health related need or disability experiencing problems with their participation. This is a middle range theory. The term ‘middle range’ theory refers to the level of abstraction at which useful theory for realist work is written: detailed enough and ‘close enough to the data’ that testable hypotheses can be derived from it, but abstracted enough to apply to other situations as well [26]. Middle range is useful because it offers an analytical approach to linking findings from different situations [26]. The outcomes of a realist review are ideally framed as middle range theory—that is, theory that can usefully be applied across a range of situations, or in a number of domains [26]. Findings are (by design) age limited (4–12 years old), but are independent of gender, disability category or ethnicity, supporting application across a range of clinical and educational settings. Ideas reflect a contemporary conceptualization of participation drawn from 72 research papers. The model imagines mechanisms and contexts in dynamic and transactional relationships. This is a “generative causality” model. Explanation is not a matter of a singular mechanism or a combination of mechanisms asserting influence on an outcome. School participation emerges out of a cooperation of factors.

No single factor fully explains variance in participation [56]. Previous research provides indications of which features of the child significantly affect the participation of students with disabilities, including focus on psychosocial factors for participation, such as preferences [102]. Our findings support the significant importance of children’s preferences, interests and motivations for participation. Our review also adds to the literature by providing detail on habits and routines which are novel elements not commonly considered. Based on our findings, we recommend that issues associated with habits and routines are closely considered in future. We have found that deficits in routine and habits are important contributors. Habit and routines are performed repeatedly and are relatively automatic. They specify what the child will do and in what order, and, thus, constitute key mechanisms for participation. Habits and routines must be understood and internalized and there are additionally ties to environment. As noted by Engman and Cranford (2016), the quality of habitual action is not equally easy for all—in some environments “non-normative embodiment” (i.e. disability) is less likely to make habitual behaviour achievable than in others [104]. The structure of the environment enables or restricts consistent, structured and planned schedules and routines. Adults facilitate breaks, social routines, setting of rules and expectations, while objects (timetables and other prompts) provide specific routines (e.g. for gathering information, or which classes to go to).

Our model focusses on participation as a key outcome which is influenced by environmental factors. In line with the ICF [1], and in the wider literature, the environment is noted to have a significant influence on participation [8, 75, 78, 92, 100]. We advance thinking by identifying specific environment factors and offering potential for comprehensive assessment and intervention. This is important, as the potential selection of environmental factors is vast. The issue is to identify specific matters facilitating or obstructing participation in school. The identification of issues must be completed in tandem with a contemporary model of participation itself. Small aspects of the school microsystem can go unnoticed if attention is not drawn to them. A focus on the school environment explicitly defined will support guidelines for working to support participation. The current study findings indicate contextual influence of the school is not just a sum of the people, objects and spaces, but also “how” things are done, or expected to be done within the school (the structures and organization of the school) and the important part adults play in providing opportunities for participation and social roles. Our findings highlight the importance of a nuanced understanding of the environment and not just consideration of physical aspects. Identification of physical aspects of the school, whist important, should always be considered alongside the social environment.

### Implications for practitioners

International practice is moving towards the adoption of system/ecological views, but the field still operates predominantly from a unidirectional perspective where “something” is provided to “fix” the person with a disability [18], rather than operating from more contemporary view of participation as a phenomenon that can be mobilized at different levels. The findings of this review show that individual and environmental interventions should be developed promote participation outcomes in schools. Identified mechanisms offer a potential basis for developing psychosocial child-focused interventions. Mechanisms (e.g. preferences, perceptions of self, perceptions of roles, internalization of routines) are appropriate targets for intervention. These ideas are congruent with recent studies emphasizing that individually tailored coaching and mentoring may help to improve children’s participation [17]. As noted, however, change will not be effective if it is only targeted at the child. Contextual elements interact with mechanisms to make participation more or less likely and must also be a focus for intervention.

With a focus on school, teachers’ knowledge is of key importance [105]. Efforts are required to assist teachers’ regarding knowledge and confidence in enhancing participation. Teachers work with increasingly diverse groups of learners and are responsible for attempting to achieve positive outcomes [106]. Concerns have been expressed that education remains less effective for learners with disabilities [2]. Concerns are understandable particularly when schools and teachers tend to be rated on achievement, rather than participation [107]. Existing supports, strategies and approaches for children with disabilities, along with theoretical underpinnings, are frequently superficial and lacking in detail [108]. Practical aspects of how to “do” inclusion or participation are therefore difficult to see and implement. Previously developed supports and interventional resources have also tended to focus on specific issues or diagnoses (e.g. Autism, Dyslexia, Learning Disability)–leading to “a programme for every problem” [109]. This has two consequences. Firstly, educationalists follow a medical or disease orientated model, with the consequential issues around disempowerment and depersonalization of people with disabilities [18]. Secondly, those with responsibility for supporting children with disabilities may feel overwhelmed by the range of options [105]. The complexity and number of programs makes selecting the right option for the right child at the right time difficult.

### Implications for research

Future research could explicitly link intervention components to mechanisms as described in this review. Following methods which use formal means for developing theoretically determined interventions [110], ‘theory-based’ rather than ‘theory-inspired’ interventions, may be developed. Such research is closely aligned to the UK MRC framework for development of complex interventions [27]. Identified mechanisms offer a basis for understanding how and why therapeutic or educational interventions for children may or may not be effective at improving school participation. Identification of strategies for the detection and cultivation of facilitative contextual elements would also follow from the above methods.

Further research activities include selection of appropriate items for school participation measurement. Parent-report methods have been commonly used in medical and psychological research to collect participation information [86]. However, researchers should also consider other data collection methodologies, particularly report by teachers [19].

### Limitations

While we have attempted to make our search as sensitive as possible (and erred on the side of sensitivity as opposed to specificity), participation continues to be a diverse area spanning several disciplines with limited consensus on terminology. It is difficult to design a perfect strategy. Given the methodological assumptions of realism, other reviewers could come to different conclusions. However, themes and concepts driving the model were apparent across different types of difficulties/disabilities, across studies that used different research methods, and across a range of international contexts. Consistency in identified features provides evidence to support conclusions.

## Conclusions

This was the first realist review to explore mechanisms and contexts for school participation of children with disabilities. This paper presents a conceptual framework including child psychosocial factors, such as understanding of routines, sense of self, and perceptions of role, and as well as characteristics of the school environment. We encourage researchers, practitioners, and policymakers to consider these contexts and mechanisms when addressing school participation among children with disabilities. Consideration of interventions, designed specifically to enhance participation by targeting mechanisms, contexts and the processes identified in this review, is now key.

## Supporting information

S1 File Appendices. (DOCX)

S2 File PRISMA standards. (DOC)

S3 File RAMESES standards. (DOCX)
