# Supplementary material for: Participation of children with disabilities in school: A realist systematic review of psychosocial and environmental factors
Source: PLoS One. 2019 Jan 29;14(1):e0210511. doi: 10.1371/journal.pone.0210511 (PMC6350972; doi:10.1371/journal.pone.0210511)
Supplement: S1 File — (DOCX) [file pone.0210511.s001.docx]

**Appendix A**

**Appendix A: Sensitizing literature review for development of initial theory**

| Reference | Key ideas |
| --- | --- |
| Adair et al., 2015^1^ | The authors explore how health, psychological, and educational interventions can improve the participation of children with disabilities in activities in the contexts of home, school, and the community. They argue that only 3 studies had participation outcomes as a primary focus, 4 used participation measures as part of a broader study – which suggests there is limited research about participation outcomes and no consistent measurement was used across studies. The authors question the WHO definition of participation and suggest the need to consider and report on operational definitions of participation in future research. Findings from a small number of studies, using different measures suggest that interventions targeting body function and activity did not improve participation but that individually tailored programmes with a goal of improving aspects of participation were more effective in enhancing participation. |
| Anaby et al., 2013^2^ | The paper provides an evidence about the influence of environment on participation, for children aged 6-14 years with physical disabilities (74% of studies) and with other disabilities. The results suggest that the attitudes domain is the leading barrier for all disability groups. The authors also suggest that conceptualizing environmental supports and barriers as opposites is unhelpful and advise grading the level of support and level of interference when measuring environment. |
| Bronfenbrenner, 1979^3^ Bronfenbrenner & Morris, 1998^4^ | The microsystem is the system closest to the person and the one in which they have direct contact. Some examples would be home, school, day care, or work. A microsystem typically includes family, peers, or caregivers. Relationships in a microsystem are bi-directional. In other words, your reactions to the people in your microsystem will affect how they treat you in return (Bronfenbrenner’s ecological model). Understanding the individual is a complex process. Bronfenbrenner stated that to understand the child, the environment in which the child lives must be fully examined. Bronfenbrenner later stressed "person-context interrelatedness." |
| Dijkers, 2010^5^ | This review explores a number of major issues related to the conceptualization, operationalization, and measurement of participation. It argues that defining and operationalizing participation requires developers of measures to consider these choices as a package. Given the complexity of participation and its manifestations in various groups defined by age, culture, and accessibility of the physical and sociocultural environment, it will be far from easy to create a measure that is simple, yet reflects participation adequately based on the conceptualization of most stakeholders. However, review concludes that improvements over the currently available instruments are feasible and needed, and further suggests that quantification of participation should be sought using the following orienting questions: What is the definition of participation? Where is the border between Participation and Activity? Is there more to participation than performance? What domains should be included in a participation measure? What are the appropriate metrics in quantifying participation? How do we define adequate participation? How should participation be operationalized? What is the proper measurement model for participation instruments? How should we collect data on participation? How do we evaluate the quality of a participation instrument? |
| Imms et al., 2016^6^ | This paper provides clarity over the definition and use of the term” participation”. The authors provide a critique of the WHO definition of participation and the resultant challenges. They investigated how researchers resolve the issue of participation as a complex construct when designing and reporting intervention research in childhood disability. Five themes were identified as predictors of participation (preferences; attendance and involvement; activity competence and sense of self). However, there was a striking lack of definitional clarity in relation to the participation construct. Along with the lack of clarity in the definition of the participation construct, this review found a mismatch between the language and the measures used. A model is proposed and authors advise that it is critical that a consistent distinction is made between the actual participation phenomenon and related concepts. The participation experience consists of two elements: attendance and involvement, with attendance being a necessary prerequisite to involvement. The related concepts (preference, activity competence, and sense of self) are also important, as is evident by their frequent inclusion in participation literature, but they are distinct from the participation phenomenon: they are not essential to any particular participation experience. |
| Kielhofner , 2007^7^ | MOHO is interested in fit between the person and their environment and the impact this has on a person’s engagement in daily living activities. In MOHO, the environment is composed of spaces (e.g. ambient, architectural and interior design features), objects (material objects and physical resources), task structure & organization (the “way” something is done, e.g. how to line up) and groups/relationships (the other human beings in the environment and their collective influence). MOHO theorises participation emerges via a combination of factors including values, interests, sense of capacity and efficacy, role performance, habits and skills, and that the environment influences behaviours and performance by providing resources and constraints. |
| Magasi et al., 2015^8^ | The review provides a critique of the ICF. It suggests that given the complex nature of environmental factors (EF) and their influence on participation, there is a need for a fresh approach to EF measurement. It suggests that the thoughtful application of theories and the use of advanced psychometric measurement, and e-technologies and data visualization methods may enable researchers and clinicians to better quantify, document, and communicate the dynamic interrelationship between EFs and participation, and health outcomes for people with disabilities at the individual, group, and population levels. |
| Maxwell & Granlund, 2011^9^ | Conditions for participation are easy to express as available, accessible opportunities, or affordability issues, but not as involvement experiences linked to accommodations made and acceptability issues within a context. Documents in Scotland and Sweden also have different foci in terms of conditions for participation. National documents in both Scotland and Sweden contain a higher proportion of references to participation conditions than those found at other levels. Differing social and cultural attitudes can clearly be seen in the Scottish and Swedish documents. |
| Maxwell et al., 2012^10^ | The paper examines the role of participation and the ICF/ICF-CY in educational research. It offers a spectrum model for the relationship between participation and environment – including 5 environment dimensions in relation to frequency and intensity of participation/ activity. It adds further evidence of the way researchers conceptualize environment and participation. It further concludes that frequency of attending and doing an activity are strongly related to the availability and accessibility of the environment, while the degree of involvement while being there is strongly related to how well adapted and acceptable the activity is for the child and others in the close environment. |
| Pratt et al., 2008^11^ | The article examines the factors that influence participation of children with cerebral palsy. It concludes that besides personal, physical function and environmental accessibility which are well examined, other factors such as personal and social motivating factors, use of community resources when available, goals setting for the child and family, and the qualities of the environments in which the child functions are not very well represented and studied. |
| Rochette et al., 2006^12^ | This review provides a reflective look at the concept of participation and the meaning of ‘optimality’ for individuals with and without disability. It suggests that “optimal” participation would rely on a perfect fit between an individual's reality (how activities and roles are actually realised) and expectations of how activities and roles should be accomplished. A transition period, including a response shift, following an acute event or onset of a chronic condition can lead to an optimal participation level despite persisting disabilities. The study concludes that interventions aimed at optimizing participation through assisting clients who are experiencing a response shift can then be designed to maximize participation and concomitantly, quality of life in those with chronic health conditions. |
| Solli et al., 2012^13^ | The study aims to analyze the ICF’s claim to holism. The following components of the ICF’s complexity are analyzed: (1) health condition, (2) body functions and structures, (3) activity, (4) participation, (5) environmental factors, (6) personal factors, and (7) health. The authors argue that although the ICF claims to be holistic, it presupposes a monistic materialistic ontology. They further indicate some limitations of this ontology, proposing instead: (a) a pluralistic–holistic ontology (PHO) and (b) a multidimensional view of the human being, with individual and environmental aspects, in relation to three levels of reality implied by the PHO. The authors conclude that for the ICF to attain its holistic claim, the interactions between its components should be based on (a) and (b). |
| Warda, 1992^14^ | This article explores the presence of chronic sorrow in families of chronically ill children and applies role theory concepts in the understanding of this phenomenon. Research findings in the area of chronic sorrow**,** family adaptation, and role theory are used to formulate propositions specific to the application of role theory in the study of chronic sorrow. The clinical and research applicability for nurses working with families of chronically disabled children are discussed to assist in providing quality family**-**nursing care. |
| Whiteneck & Dijkers, 2009^15^ | This review addresses conceptual and methodological issues related to the ICF constructs, and recommends an improved distinction between activities and participation, and further elaborates about the environment. It argues that environment is very broad and we cannot begin to measure all aspects of it simultaneously. It concludes that we need to limit our quantification to those aspects of the human-made, natural, social, and cultural environment that potentially have a role in limiting or enhancing the functioning of individuals with defined impairments. To do so, we need a theory of how environments affect functioning, however unsophisticated that theory (or collection of theories) may be. Once the theory has led us to the parts or aspects that are presumed to be relevant, we need an instrument or instruments to measure these. It further argues that the development of proper instruments for operationalizing environments may require us to abandon classic test theory and related measurement models assuming latent traits that can be quantified using effect indicators. |
| WHO (2001)^16^ | Participation is involvement in a life situation. Participation restrictions are problems an individual may experience in involvement in life situations. Activity is the execution of a task or action by an individual. Activity limitations are difficulties an individual may have in executing activities. Performance is what an individual does in the current environment, and capacity is an individual's maximal ability in an ideal or standardized environment. |

**Appendix B**

**Appendix B: Example Search strategy**

**Database: Medline (EBSCOhost)**

**Search strategy: disability/age/ participation/ environment/ school**

S1 (MM "Disabled Persons+")

S2 (MM "Motor Skills Disorders")

S3 (MM "Developmental Disabilities")

S4 (MM "Intellectual Disability+")

S5 (MM "Communication Disorders+")

S6 (S1 OR S2 OR S3 OR S4 OR S5)

S7 (AB disab* OR AB special N1 needs OR TI disab* OR TI special N1 needs)

S8 (S6 OR S7)

S9 (MM "Child+")

S10 (MM "Pediatrics+")

S11 (TI child* OR TI girl* OR TI boy* OR TI schoolchild* OR AB child* OR AB girl* OR AB boy* OR AB schoolchild*)

S12 (S9 OR S10 OR S11)

S13 (MM "Social Participation")

S14 (TI participation OR TI inclusion OR TI engagement OR TI "life N2 situations" OR AB participation OR AB inclusion OR AB engagement OR AB "life N2 situations")

S15 (S13 OR S14)

S16 (MM "Social Environment+")

S17 (TI environ* OR TI surround* OR TI setting* OR TI built* OR TI context* OR AB environ* OR AB surround* OR AB setting* OR AB built* OR AB context*)

S18 (S16 OR S17)

S19 (MM "Schools+")

S20 (TI school* OR TI education* OR TI class* OR TI teach* OR AB school* OR AB education* OR AB class* OR AB teach)

S21 (S19 OR S20)

S22 (S8 AND S12 AND S15 AND S18 AND S21)

S23 (S22 limiter –dates 2006-2018)

S24 (S23 limiter-language English)

**Appendix C**

**Appendix C: Characteristics, participation outcomes and limitations of eligible studies in the main review**

| Reference and  Setting | Design | Sample (n) | Children’s Mean Age, Y:mo  (SD), Range | Participation | Critical appraisal issues |
| --- | --- | --- | --- | --- | --- |
| Egilson & Traustadottir, 2009a^17^  (Iceland) | Qualitative  Grounded Theory Study | Children with physical disabilities (14: 9 boys, 5 girls)  Parents (17)  Teachers (18) | 6-12 | Attendance | Sample size  Data collection: observation of one school day  Follow up: parents interviews |
| Nespor et al., 2009^18^  (USA) | Qualitative  Institutional Ethnographic Study (part of a larger cross-generational study) | Parents (24) reported on children with complex cognitive and physical disability | 6 | Attendance | Sample size |
| Reszka et al., 2012^19^  (USA) | Quantitative  Descriptive Study  (part of a larger ASD treatment Comparison Study) | Children with ASD (68: 58 boys, 10 girls) | Mean chronological age 3.86 (SD=0:54) | Attendance | Data analysis: data coded from videotaped samples |
| Specht et al., 2011^20^  (Canada) | Qualitative  Phenomenological Exploratory Study | Teachers (15: 3 men, 12 women)  Children (24: 14 boys, 10 girls)  Children with disabilities (9: 5 boys, 4 girls) | Grade 4-7 | Attendance Involvement | Sample size  Data collection: teacher description, children observation |
| Spencer-Cavaliere & Watkinson, 2010^22^  (Canada) | Qualitative  Idiographic, Exploratory and Descriptive Design | Children with disabilities (11: 9 boys, 2 girls) | 10:5, 8-12 | Attendance Involvement | Sample size  Sample: half of the sample have CP(little variance, shared experience)  Sample: unequal representation of genders |
| Woolfson et al., 2007^23^  (Scotland) | Mix-Methods  Sequential Explanatory Study | Children with disabilities (98)  Children with disabilities (30: focus group) | 9 -14 | Attendance | Data collection: authenticity of the findings (child opinion vs. parent)  Data collection: not all children engage during focus group |
| Mei et al., 2015^25^  (Australia) | Qualitative  Phenomenological Study | Parents (13) of children with CP | 4-9 | Attendance | Sample size  Methodology: no verification of results with participants |
| Colver et al., 2012^26^  (Europe) | Quantitative  Cross Sectional European Study | Children with CP (818: 483 boys, 335 girls) | 7-13 | Attendance | Design: cross-sectional, unable to determine causation |
| Thornton et al., 2015^28^  (Australia) | Quantitative  Quasi -experimental study | Children with DCD (20 boys) | 9:1 (0:9), 8-10 | Attendance Involvement | Sample: limited to boys  Follow-up: none |
| Michelsen et al.,2009^29^  (Europe) | Quantitative  Cross Sectional European Study | 3,757 = children from the General Population (2939) Children with CP (813) and their parents | 7-14  7-13 | Attendance | Bias: participation of responders might be systematically different from that of non-responders |
| Field et al., 2015^30^  (Canada) | Consensus Study using a modified Delphi Survey | Parents (14) reported on 15 children who use PM  Therapists (40)  Researchers (20) reported on children with disabilities | 3-11,  18m-5y, 6-12 | Attendance Involvement | Sample: uneven representation of participants |
| Furtado et al., 2015^31^  (Brazil) | Quantitative Population Based Study | Parents of children with CP (102: 54 boys, 48 girls)  Teachers | 6-17 | Attendance | Sample: classified in levels I, II and III of the GMFCS excluding children with higher motor impairments |
| Bedell et al., 2011^32^  (USA and Canada) | Qualitative Descriptive Study | Parents of children with disabilities (25) from USA and Canada  Parents of children without disabilities (17) from USA | 5-18 | Attendance Involvement | Sample sizes  Data collection: different formats across two settings  Sample: excluding parents of children without disabilities from Canada |
| Kolehmainen et al., 2015^33^  (UK) | Mixed-methods Intervention Development Study | Parents (152) of children with motor impairment  Therapists reported for children with motor impairment (195)  Motor impairment (17) | 6-8 | Attendance Involvement | Bias: self-selection bias |
| Hwang et al., 2015^34^  (Taiwan) | Quantitative  Cross Sectional Study | The FUNDES-Child Child and Family Follow-up Survey (CFFS) dataset Taiwan  Parents (18,119) of children with disabilities | 6-17:9 | Attendance |  |
| Houtrow et al., 2012^35^  (USA) | Quantitative  Cross Sectional Study | The 2007 National Survey of Children’s Health (NSCH) 64,076 children (weighted)  Children with special health care need (17,254) Children without special health care needs (49,027) | 6-17 | Attendance | Design: cross-sectional, unable to determine causation  Choice of variables restricted to those available in the NSCH data set  Measures of health status and functional limitations are subjective |
| Egilson & Traustadottir, 2009b^36^  (Iceland) | Mixed-methods design using a Grounded Theory approach. | Children with physical disability (14: 9 boys, 5 girls)  Parents (17)  Teachers (18)  Parents (32) of children with disabilities (32: 14 boys, 18 girls) | 6-12 | Attendance Involvement | Sample sizes |
| Peny-Dahlstrand et al., 2013^37^  (Sweden) | Quantitative  Population Based, Cross Sectional and Exploratory. | Children with SB (50: boys 26, 24 girls)  Teachers (48) | 10:5, 6-14 | Attendance | Data collection: didn’t include children’s views on their level of active involvement and their feelings of belonging  Data collection: influence of environment of participation was not considered |
| Wight & Chapparo, 2008^39^  (Australia) | Quantitative Correlational Pilot Study | Children with LD (21 boys)  Comparison group (21 boys) | 5-11 | Attendance Involvement | Sample size  Sample: limited to boys |
| Raghavendra et al., 2012^38^  (Australia) | Quantitative  Cross Sectional, Matched Multi Group, Comparison Study | Children with physical disability and communication (14:8 boys, 6 girls)  Physical disability (11: 7 boys, 4 girls)  Typical peers (14: 8 boys, 6 girls) | 10-15 | Attendance | Small sample size  Data collection: observation without videotaping |
| Parslow et al., 2015^41^  (UK) | Qualitative Exploratory Design | Children with CFM/ME (25: 11 boys, 14 girls) | 12:9 (2:2), 8-17 | Attendance Involvement | Data collection: parents presence during interview  Sample recruitment |
| Sentenac et al., 2013^42^  (Europe) | Quantitative  Cross Sectional European Study | Children with CP (818) | 8-12 | Attendance | Analysis: Italy and Germany were not included in multilevel analysis  Analysis: grouping children from special units in mainstream school with ones in special school |
| Dolva et al., 2011^43^  (Norway) | Qualitative Phenomenological Design | Children with Down syndrome (6: 4 boys, 2 girls)  Teachers (6)  Teacher assistants (6) | 10 | Attendance  Involvement | Sample size  Measurement: no data on frequency of interactions among children |
| Yalon-Chamovitz et al., 2006^44^  (Israel) | Quantitative Exploratory and Correlational study | Children with mild LD ( 30: 18 boys, 12 girls) | 7-15 | Attendance | Sample size  Data collection: observation |
| Eriksson et al., 2007^46^  (Sweden) | Quantitative Exploratory and Correlational study | Children with disabilities (33: 16 boys, 17 girls)  Typical peers (33: 17 boys, 16 girls ) | 9:9 (1:87), 7-12 | Attendance | Sample size  Data collection: each child was observed during one school day |
| Almqvist, 2006^47^  (Sweden) | Quantitative Exploratory and Descriptive Study | Children with developmental delay and typical peers (1035: 52.6% boys, 47.4% girls ) | 14-68 months | Attendance | Methods: a large number of instruments used which were partly modified, and their construct validity was not obtained. Data collection: large dropout in parent ratings |
| Rosenberg et al., 2012^48^  (Israel) | Quantitative Descriptive Correlational Study | Children with mild developmental disability (78: 63 boys, 15 girls) | 5:20 (0:52), 4-6 | Attendance Involvement | Sampling method: data collected from community paediatric clinics  Potential bias: study measures were developed by the same team |
| Rosenberg et al., 2013^49^  (Israel) | Quantitative  Quasi -Experimental Study | Children with mild developmental disability(78: 63 boys, 15 girls  Typical peers (110: 76 boys, 34 girls) | 5:27 (0:60), 4-6 | Attendance Involvement | Measurement: 5 dimensions of participation  Evaluators: not blind to the study purpose |
| Pereira et al., 2010^50^  (Portugal) | Qualitative Phenomenological Study | Children with disabilities (14) | 8-11 | Attendance Involvement | Sample size  Selection of participants: all with good communication skills  Data collection: interview  Methodology: methodological triangulation was decreased as only first author speaks Portuguese |
| Pivik, 2010^51^  (Canada) | Quantitative  Multi-Site Descriptive Study | Schools with children with disabilities (29)  Schools with typical peers (22) | 13 (2:9)  13 (0:48) | Attendance | Data collection: lack of methodological control  Students selection: principal- lead |
| Barnett, 2013^52^  (UK) | Qualitative Exploratory and Descriptive Study | Children with DCD (8:8 boys)  Parents (8) | 13-15 | Attendance Involvement | Sample size  Sample: limited to boys |
| Law et al., 2011^53^  (Canada) | Quantitative  RCT Cluster Research Design | Children with CP (128: 79 boys, 49 girls) | 3:6 (1:5), 12months-5:11 | Attendance | Findings: inability to extrapolate to typical therapy services |
| Coster et al., 2013^54^  (USA and Canada) | Quantitative  Cross Sectional Descriptive Comparison Study | Parents of children with disabilities (282:166 boys, 116 girls)  Typical peers (294: 145 boys, 149 girls) | 11:7 (3:106), 5-17  10:6 (2:975), 5-17 | Attendance | Data collection: online internet survey  Sample: no random selection |
| Williams et al., 2015^55^ (Ireland) | Quantitative Population based, correlational study | Parents of children with ASD (109: 80 boys, 29 girls) | 9:74 (3:17), 6-17 | Attendance | Data collection: parent-report  Bias: response bias |
| Logan et al., 2015^57^  (USA) | Quantitative Subjects repeated measures cohort design | Children with neuropathic or musculoskeletal pain (274: 16% boys, 84% girls) | 14:6 (2:3),10-18 | Attendance | Data collection instrument: initially developed for use in school age children  Data collection: self-reported measurement  Sample: mostly extremity-based pain condition  Potential bias: missing data across time points |
| Logan et al., 2012^56^  (USA) | Quantitative  Cross Sectional Descriptive Study | Children with chronic pain (350: 19.4% boys, 80.6% girls) and their parents | 13:7 (2:5), 8-17 | Attendance | Sample: parental participation limited to mothers  Sample: mostly girls, parent-child dyads might be an issue  Data collection: limitations to the way school attendance data were collected  Design: cross-sectional, unable to determine causation |
| Matsuoka et al., 2014^58^  (Japan) | Quantitative  Cross Sectional School Based Survey | Children with developmental disorder (43: 39 boys, 4 girls)  Typical peers (372: 182 boys, 190 girls) | 9:1 (1:5), 6-12  9:4 (4.5), 6-12 | Attendance Involvement | Data collection instrument: reliability and validity of Japanese version of CSHQ has not been determined yet  Sample: % of children being on medication in the DD group was significantly higher compared to the control  Results: subjective data |
| Maher et al., 2015^60^  (Australia) | Quantitative  Cross Sectional Study | Children with mild and moderate physical disabilities ( 65: 35 boys, 30 girls) | 13:2 (2:8), 8-17 | Attendance | Sample size, single city  Sample: only children with mild and moderate physical disabilities  Data collection: additional factors that may be associated with fatigue were not measured  Design: cross-sectional, unable to determine causation |
| Foster-Cohen & Mirfin-Veitch, 2015^62^  (New Zealand) | Qualitative Programme Evaluation Study | Parents (21)  Teachers (21)  Children with developmental disability (23: 19 boys, 4 girls) | 5:7-11:10 | Involvement | Sample size  Design: qualitative, no control group, range of variables can impact the outcome |
| Soref et al., 2012^63^  (Israel) | Quantitative Correlational Study | Children with mild motor disability (29: 23 boys, 6 girl)  Typical peers (29:23 boys, 6 girls) | 5:14 (0:46), 4:50-5:92  5:28 (0:57), 4:5-6 | Attendance Involvement | Sample size  Sample: demographic limitation, most from urban communities  Data collection: limited to investigation of maternal factors |
| Colver et al., 2011^64^  (Europe) | Quantitative  Cross Sectional European Study | Children with CP (818: 484 boys, 334 girls) | 8-12 | Attendance | Bias: participation of responders might be systematically different from that of non-responders |
| Berrin et al., 2007^65^  (USA) | Quantitative  Cross Sectional Study | Parents (189) reported for children with spastic CP  Children with spastic CP who self-reported (73) | 8:4 (4:4), 5-18  10:3 (3:9), 5-18 | Attendance | Sample: excluded children with mental retardation and Down’s syndrome  Design: cross-sectional  Data collection: no data were gathered on intellectual ability |
| Badia et al., 2014^66^  (Spain) | Quantitative  Cross Sectional Study | Physiotherapists (35) reported on  CP( 91: 41 boys, 50 girls) | 12, 8-19 | Involvement | Data collection: limited to physiotherapists  Sample size  Sample: CP with different level of cognitive impairment  Design: cross-section, unable to determine causation |
| Fauconnier et al., 2009^67^  (EUROPE) | Quantitative  Cross Sectional European Study | Children with CP (818: 484 boys, 334 girls) | 8-12 | Attendance | Bias: participation of responders might be systematically different from that of non-responders |
| Chen et al., 2014^68^ (Chile) | Qualitative Descriptive Study | Parents (9) of children with disabilities  Rehabilitation providers (18) | 8:5, 6-13  6:3, 1-12 | Attendance | Sample size  Bias: participant volunteers might be more knowledgeable than nonparticipants |
| Parslow et al., 2017^69^ (UK) | Qualitative Descriptive Study | Health professionals (15) report on children with CFM/ME | 2months-25 | Attendance Involvement | Sample size  Data collection: limited to health professionals |
| Piškur et al., 2017^70^ (The Netherlands) | Qualitative Descriptive Study | Parents (47) of children with physical disabilities | 4-12 | Attendance Involvement | Sample size  Data collection: limited to caregivers |
| Øien et al., 2016^71^  (Norway) | Qualitative Descriptive Study | Children with CP (9) | 5-6 | Attendance Involvement | Sample size  Data collection: observations |
| Ravenscroft et al., 2017^72^ (Spain) | Quantitative  Cross Sectional European Study | Parents (306) of children with complex additional support | 3-13 | Attendance | Data collection: language bias, selection bias |
| Chien et al., 2017^73a^ (Australia) | Quantitative | Parents (58) of children with moderate-to-severe developmental disabilities | 2-12 | Attendance Involvement | Sample size  Data collection: limited to caregivers, limited number of predictors  Design: cross-section, unable to determine causation |
| Chien et al., 2017^74b^ (Australia) | Quantitative | Parents of 64 (42 boys:22 girls) children with moderate-to-severe developmental disabilities | 2-12 | Attendance Involvement | Sample size  Data collection: limited to caregivers, limited number of predictors  Design: cross-section, unable to determine causation |
| Costa et al., 2017^75^ (Austria) | Mixed-method | Children with disabilities (38)  Parents (32)  Teachers (19) | 5-10 | Attendance Involvement | Sample size |
| Jindal et al., 2017^76^ (India/Canada) | Qualitative Descriptive Study | Parents of children with CP from India (11) and Canada (7) | 2-10 | Attendance | Sample size  Data collection limited to English speaking caregivers |
| Earde et al., 2018^77^  (Thailand) | Qualitative Descriptive Study | Parents of children with cerebral palsy (27) | 4-12 | Attendance Involvement | Sample size  Data collection: limited to caregivers |

**Appendix D**

**Appendix D: Characteristics and limitations of eligible review studies in the main review**

| Reference and  Setting | Title | Review type  (time span) | No. articles included | Critical appraisal issues |
| --- | --- | --- | --- | --- |
| Maxwell & Granlund, 2011^9^  (Scotland and Sweden) | How are conditions for participation expressed in education policy documents? A review of documents in Scotland and Sweden. | Policy documents review  (1993-2010) | 41 | Data collection: limited selection of policy papers  Data synthesis: restricted to 2 researchers, novel use of coding methodology |
| Simpson et al., 2011^21^  (USA) | Critical Educational Program Components for Students with Emotional and behavioral Disorders: Science, Policy, and Practice. | Narrative review  (Not reported) | Not reported | No search strategy |
| Hume & Reynolds, 2010^24^  (USA) | Implementing work systems across the school day: Increasing engagement in students with autism spectrum disorders. | Narrative review  (Not reported) | Not reported | No search strategy |
| Lindsay, 2016^27^  (Canada) | Child and youth experiences and perspectives of cerebral palsy: A qualitative systematic review. | Systematic review/  Qualitative synthesis  (1980-Sepetember, 2014) | 33 | Data synthesis: wide age range among study participants |
| Pratt et al., 2008^11^  (USA) | Participation of the child with cerebral palsy in the home, school, and community: A review of the literature. | Literature review  (1991-2008) | 34 | Publication date  Data synthesis: incorporated studies that include children with other physical disabilities |
| Webster & Carter, 2007^40^  (Australia) | Social relationships and friendships of children with developmental disabilities: Implications for inclusive settings. A systematic review. | Systematic review/  Qualitative synthesis  (Not reported) | 36 | Publication date  Data synthesis: in many cases literature did not clearly specify population under investigation; limited focus on inclusive setting |
| Anaby et al., 2013^2^  (Canada) | The effect of the environment on participation of children and youth with disabilities: A scoping review. | Scoping review  (1990-2011) | 31 | Inclusion criteria: focus on out-of-school participation  Data collection: no search of grey literature  Data synthesis: no quality assessment of studies under review |
| Snyder et al., 2015^45^  (USA) | Naturalistic instructional approaches in early learning: A systematic review. | Systematic review/  Qualitative e synthesis  (1980-2013) | 43 | Inclusion criteria: limited to NI approaches in preschool setting |
| Carroll et al., 2015^59^  (UK) | Understanding fatigues in paediatric multiple sclerosis: a systematic review of clinical and psychosocial factors. | Systematic review/  Narrative synthesis  (No date limits) | 12 | Data synthesis: lack of published literate on fatigue in MS; small sample sizes in published literature; most studies were cross-sectional |
| Adair et al., 2015^1^  (Australia) | The effect of interventions aimed at improving participation outcomes for children with disabilities: a systematic review. | Systematic review  (2001-2013) | 7 | Data collection: no specific definition of participation was utilized which impacted on interpretation of extracted data  Exclusion criteria: excluded studies that included on-task behavior as participation measure –limited number of papers from education field |
| Granlund et al., 2012^61^  (Sweden) | Differentiating activity and participation of children and youth with disability in Sweden: a third qualifier in the International Classification of Functioning, Disability, and Health for Children and Youth? | Review  (Not reported) | Not reported | Results/Discussion: founded on correlational studies and secondary analysis, thus, can only be seen as preliminary support for the conclusions drawn  No search strategy |
| Imms et al., 2016^6^  (Australia) | ‘Participation’: a systematic review of language, definitions, and constructs used in intervention research with children with disabilities. | Systematic review/  Quantitative and qualitative synthesis  (2001-2013) | 25 | Data synthesis: did not extract and analyze texts from included studies that describe environmental supports and barriers to participation  Inclusion criteria: search limited to 2013 |
| Magasi et al., 2015^8^  (USA) | "Theoretical foundations for the measurement of environmental factors and their impact on participation among people with disabilities." | Narrative review  (Not reported) | Not reported | No search strategy |
| Maxwell et al., 2012^10^  (Sweden) | Participation and environmental aspects in education and the ICF and the ICF-CY: Findings from a systematic literature review. | Systematic review  (2001-2009) | 23 | Data synthesis: results relating to personal factors component of the ICF/ICF-CY was not reported in the present review due to their lack of standardization and conceptual clarity |
| Whiteneck & Dijkers, 2009^15^  (USA) | Difficult to measure constructs: Conceptual and methodological issues concerning participation and environmental factors. | Narrative review  (Not reported) | Not reported | No search strategy |
| McGarty et al., 2018^78^  (UK) | Parental perceptions of facilitators and barriers to physical activity for children with intellectual disabilities: A mixed methods systematic review | Mixed methods systematic review (1946-2017) | 10 | Data analysis: meta-ethnographical approach |
| Bakanienė et al., 2018^79^  (Lithuania) | Participation of Children with Spina Bifida: A Scoping Review Using the International Classification of Functioning, Disability and Health for Children and Youth (ICF-CY) as a Reference Framework. | Scoping review  (1990-2018) | 10 | Data synthesis: quality of studies was not evaluated.  Results: limited by the relatively small number of primary research studies. |
| Anaby et al., 2018^80^  (Canada) | Recommended practices to organize and deliver school‐based services for children with disabilities: A scoping review. | Scoping review  (1998-2017) | 56 | Data synthesis: quality of studies was not evaluated. Focus is limited on elementary and high school children. |

**Appendix E**

**Appendix E: Mechanisms tables**

| **Hypothesis** | **Mechanisms** | **Papers addressing** |
| --- | --- | --- |
| **Identity mechanism**  The child’s identity — mechanisms associated with thoughts and feelings the child has about themselves (e.g., believing in themselves, having confidence, understanding their roles or feeling a like a member of the school community) and perceptions of school activities (e.g. interests, preferences or perceived enjoyment) | **Preferences**: interests, perceived enjoyment, attraction to activities ^6, 19, 22, 30, 32, 36, 41, 48, 63, 71, 75, 78, 79^  **Perceptions of self**: self-esteem, self-efficacy, confidence, perceived competence, feeling of empowerment ^6, 25, 28, 33, 49, 50, 52, 63,69, 71, 75, 78, 79^  **Meaningfulness**: willingness, perceptions of satisfaction ^6, 10, 30, 36, 61,71,75,77^  **Internalization/perception of roles:** understanding and knowledge of roles, feeling like a legitimate participant, feeling included, feeling membership and school identity^20, 49, 50^  **Internalization of habits and routines:** familiarity, knowledge, preparedness, and automaticity of habits and routines^21, 24, 25, 32, 43^ | ^6, 10, 19, 20, 21, 22, 23, 24, 25, 28, 30, 32, 33, 36, 41, 43,^  ^48-50, 52, 61, 63,69, 71,75, 77-79^ |
| **Competence mechanism**  The child’s competence – mechanisms associated what the child actually does in school (e.g. following rules, showing interest, being confident, following a routine or using skills) | **Making choices:** showing initiative, being proactive, expressing interest ^6, 23, 25, 32, 36, 38, 44,72,79^  **Autonomy/Responsibility/Persistence:** independence, self-reliance; being committed, working towards goals; perseverance^17, 25, 27, 30,^ ^37, 48, 50,72,75,76,79^  **Meeting role expectations/role scripts:** following rules and norms, fulfilling role expectations; routine performance in school roles and other roles (e.g. friendship role) ^20, 27, 37, 40, 50^  **Meeting habit and routine expectations:** having routines, following routines, having habits, doing what’s expected^21, 33^  **Organisation and planning skills:** Sequencing, concentration, memory, organization^11, 24, 33, 37, 41, 49,73,75,76,78,79^  **Motor skills**: gross and fine ^27, 29, 31, 33, 36, 37, 52, 53, 64, 66, 67,69,73,75,76,78,79^  **Communication skills** : expressive/ receptive, written, social communication ^19, 25, 36, 44, 46,73,75,76,78,79^ | ^6,11, 17,19, 20, 21, 23,24, 25, 27, 29, 30, 31, 32, 33, 36, 37, 38, 40, 41, 44, 46, 48, 49, 50, 52, 53, 64, 66, 67,69,72, 73,75,76,78,79^ |
| **Experience of body and mind mechanism (symptoms**  The child’s experience of mind and body (symptoms) – mechanisms associated with issues commonly experienced by children with disabilities in schools: pain, anxiety, mood and fatigue/tiredness. | **Pain**: cognitions, catastrophizing, withdrawal ^27, 29,^ ^36, 56, 57, 65, 66,^ ^67,79^  **Fatigue**: energy level, fluctuating symptoms, sleep disturbance, withdrawal ^27, 36, 41, 52, 59, 60, 68,69^  **Anxiety**: annoyance, frustration, anger, aggression, withdrawal ^25, 27, 39, 41, 52, 55, 58,69,75^  **Mood**: sadness, depression, withdrawal ^27, 35, 56, 58, 59,69,79^ | ^25, 27, 29, 35, 36, 39, 41, 52, 55-60, 65-68,69,75,79^ |

**Appendix F**

**Appendix F: Context (more detail)**

| **Context/Main themes** | **Opportunities and Constraints** | **Papers addressing** |
| --- | --- | --- |
| **Adults**  **OP: opportunity creator**  **AT: attitudes**  **KS: knowledge & skills**  **ST: structure** | Opportunities | ^10, 15, 19, 20, 21, 22, 26, 32, 36, 38, 43, 47, 50, 54, 70, 72, 78, 79, 80^ |
|  | OP: provide chances, shaping positive roles ^10,15, 19, 20, 22, 38, 43, 50, 54, 70, 79, 80^  KS: competent staff ^21, 32, 70, 72, 78 ,80^  AT: positive attitudes^26, 36, 47,70, 80^  ST: collaboration ^36, 47, 70, 72, 80^ |  |
|  | Constraints | ^2, 11, 17, 20, 25-27, 32, 36, 47, 48, 52, 69, 70, 76, 78^ |
|  | OP: shaping negative roles^20^  AT: unsympathetic attitudes ^2, 25-27, 32, 47, 48, 52, 69, 70, 76^  KS: lack of knowledge ^11, 36, 70, 78^  ST: poor communication between staff ^11, 17, 70^ |  |
| **Peers**  **SP: support**  **FR: friendship**  **AT: attitudes** | Opportunities | ^2, 6, 9, 10, 15, 17, 20, 22, 26, 27, 32, 40, 41, 43, 46, 47, 50, 52, 72, 77, 78^ |
|  | SP: encouraging; practical and emotional support ^2, 9, 10, 15, 22, 32, 41, 43, 46, 50, 52, 72, 77, 78^  FR: nurturing relationships; friendship ^20, 22, 27, 40,78^  AT: positive attitudes ^6, 9, 17, 26, 41, 47^ |  |
|  | Constraints | ^2, 20, 22, 25, 26, 27, 30, 32, 38-42, 46, 47, 50, 52, 64, 69, 70, 77, 78^ |
|  | SP: discouraging; negative actions; bullying; discrimination ^20, 22, 27, 30, 32, 40, 42, 46, 50, 69, 70^  FR: friendship avoidance ^27, 38, 39, 46, 77, 78^  AT: negative attitudes; stigma^2, 25, 26, 41, 42, 47, 52, 64, 69^ |  |
| **Structure & organization**  **TA: tailoring to child**  **RN: flexibility**  **ST: structure** | Opportunities | ^1, 2, 6, 9, 10, 17-24, 28, 32, 34, 36, 37, 42-45, 46, 47, 50, 52, 54, 62, 69, 72, 78-80^ |
|  | TA: equal opportunity providing^20^ responsive to needs; individualized; child mediated ^1, 2, 6, 10, 17, 19, 21-24, 28, 32, 34, 37, 42-45, 46, 47, 50, 52, 62, 69, 72, 78-80^  RN: adaptable; flexible ^2, 9, 17, 18, 23, 32, 36, 43, 54, 69, 72, 79, 80^  ST: consistency/predictability ^32^ planning and collaboration ^2, 17, 18, 47, 72, 80^ |  |
|  | Constraints | ^2, 8, 11, 17-19, 23, 25, 27, 32, 36-38, 42, 44, 46, 47, 50, 52, 54, 59, 62,^ ^69, 76, 78^ |
|  | TA: not individualized^8, 11, 17-19, 23, 25, 27, 32, 38, 46, 50, 52, 54, 62, 69, 76, 78^  RN: rigid ^2, 11, 18, 36, 42, 44, 47, 54, 59, 69^  ST: unpredictable ^37^ unstructured and/or lack of rules or regulations^37, 76^ |  |
| **Spaces**  **AV: available**  **AC: accessible**  **ST: suitable** | Opportunities | ^2, 6, 9, 10, 17, 21, 23, 25, 27, 30, 32, 36, 43, 44, 54, 61, 64, 68, 69, 72, 77, 80^ |
|  | AV: spaces exist^17, 43^  AC: usable as required ^2,6, 9, 10, 17, 23, 27, 30, 32, 36, 44, 54, 61, 64, 69, 72, 77^  ST: suitable; design “just right”’; layout “ just right”; sensory qualities attended; sensory qualities modifiable ^21, 25, 68,70,72, 77, 80^ |  |
|  | Constraints | ^2, 11, 23, 25, 27, 36, 42, 48, 51, 54, 64, 70, 72, 77, 78, 79^ |
|  | AC: inaccessible ^2, 11, 23, 25, 27, 36, 42, 48, 51, 54, 64, 70, 77 ,78, 79^  ST: crowded; unfamiliar; sensory qualities unmodified/unsuitable ^11, 23, 25, 54, 70, 77, 79^ |  |
| **Objects**  **AV: available**  **AC: accessible**  **ST: suitable** | Opportunities | ^2, 6, 9-11, 21, 23-25, 27, 32, 36, 61, 62, 70, 71, 76, 77, 79^ |
|  | AV: objects exist ^2, 6, 9-11, 21, 23, 24, 36, 61, 62, 70, 77, 79^  AC: usable; acceptable ^25, 27, 32, 36, 70, 71, 76, 77, 79^ |  |
|  | Constraints | ^2, 11, 23, 25, 30, 70, 71, 76, 77^ |
|  | AV: unavailable ^2, 23, 70,76,77^  ST: complicated; usability issues ^70,71^; cumbersome ^11, 25,71^, unsuitable; isolating ^30,71^ |  |

**Appendices references**

1. Adair B, Imms C, Ullenhag A, Keen D, Granlund M. The Effect of Interventions Aimed at Improving Participation Outcomes for Children with Disabilities: A Systematic Review*. Dev Med Child Neurol* 2015**; 57**: 1093-1104.

2. Anaby D, Hand C, Bradley L, et al. The Effect of the Environment on Participation of Children and Youth with Disabilities: A Scoping Review*. Disabil Rehabil* 2013**; 35**: 1589-1598.

3. Bronfenbrenner U. The ecology of human development : experiments by nature and design. Cambridge, MA: Harvard University Press, 1979.

4. Bronfenbrenner U, Morris P. The ecology of developmental processes. In: Damon, William, Lerner, et al (eds) Handbook of child psychology: Theoretical models of human development. 5th ed. ed. New York: John Wiley and Sons, Inc.: New York: John Wiley and Sons, Inc., 1998, p.993.

5. Dijkers MP. Issues in the Conceptualization and Measurement of Participation: An Overview. . 2010 Sep 30;91(9):S5-16. *Arch Phys Med Rehab* 2010**; 91**: S5-16.

6. Imms C, Adair B, Rosenbaum P, Keen D, Ullenhag A, Granlund M. 'Participation': A Systematic Review of Language, Definitions, and Constructs used in Intervention Research with Children with Disabilities*. Dev Med Child Neurol* 2016**; 58**: 29-38.

7. Kielhofner G. Model of Human Occupation: Theory and Application. 4th. ed. Baltimore: Lippincott Williams & Wilkins, 2008.

8. Magasi S, Hammel J, Heinemann AW, et al. Theoretical Foundations for the Measurement of Environmental Factors and their Impact on Participation among People with Disabilities*. Arch Phys Med Rehabil* 2015**; 96**: 569-577.

9. Maxwell G, Granlund M. How are Conditions for Participation Expressed in Education Policy Documents? A Review of Documents in Scotland and Sweden*. Eur J Spec Needs Educ* 2011**; 26**: 251-272.

10. Maxwell G, Alves I, Granlund M. Participation and Environmental Aspects in Education and the ICF and the ICF-CY: Findings from a Systematic Literature Review*. Dev Neurorehabil* 2012**; 15**: 63-78.

11. Pratt B, Baker KW, Gaebler-Spira D. Participation of the Child with Cerebral Palsy in the Home, School, and Community: A Review of the Literature*. J Pediatr Rehabil Med* 2008**; 1**: 101-111.

12. Rochette A, Korner-Bitensky N, Levasseur M. ‘Optimal’ Participation: A Reflective Look*. Disabil Rehabil* 2006**; 28**: 1231-1235.

13. Solli HM, da Silva AB. The Holistic Claims of the Biopsychosocial Conception of WHO's International Classification of Functioning, Disability, and Health (ICF): A Conceptual Analysis on the Basis of a Pluralistic-Holistic Ontology and Multidimensional View of the Human being*. J Med Philos* 2012**; 37**: 277-294.

14. Warda M. The Family and Chronic Sorrow: Role Theory Approach*. J Pediatr Nurs* 1992**; 7**: 205.

15. Whiteneck G, Dijkers MP. Difficult to Measure Constructs: Conceptual and Methodological Issues Concerning Participation and Environmental Factors*. Arch Phys Med Rehabil* 2009**; 90**: S22-S35.

16. World Health Organization. International Classification of Functioning, Disability, and Health: ICF. Switzerland, 2001.

17. Egilson ST, Traustadottir R. Assistance to Pupils with Physical Disabilities in Regular Schools: Promoting Inclusion Or Creating Dependency?*. Eur J Spec Needs Educ* 2009**; 24**: 21-36.

18. Nespor J, Hicks D, Fall AM. Time and Exclusion*. DISABIL SOC* 2009**; 24**: 373-385.

19. Reszka S, Odom S, Hume K. Ecological Features of Preschools and the Social Engagement of Children with Autism*. J Early Interv* 2012**; 34**: 40-56.

20. Specht JA, King GA, Servais M, Kertoy M, Spencer T. School Roles: A Way to Investigate Participation*. Except Educ Int* 2011**; 21**: 2-14.

21. Simpson RL, Peterson RL, Smith CR. Critical Educational Program Components for Students with Emotional and Behavioral Disorders: Science, Policy, and Practice*. Remedial Spec Educ* 2011**; 32**: 230-242.

22. Spencer-Cavaliere N, Watkinson EJ. Inclusion Understood from the Perspectives of Children with Disability*. Adapt Phys Activ Q* 2010**; 27**: 275-293.

23. Woolfson RC, Harker M, Lowe D, Sheilds M, Mackintosh H. Consulting with Children and Young People Who have Disabilities: Views of Accessibility to Education*. Brit J Spec Educ* 2007**; 34**: 40-49.

24. Hume K, Reynolds B. Implementing Work Systems Across the School Day: Increasing Engagement in Students with Autism Spectrum Disorders*. Prev Sch Fail* 2010**; 54**: 228-237.

25. Mei C, Reilly S, Reddihough D, et al. Activities and Participation of Children with Cerebral Palsy: Parent Perspectives*. Disabil Rehabil* 2015**; 37**: 2164-2173.

26. Colver A, Thyen U, Arnaud C, et al. Association between Participation in Life Situations of Children with Cerebral Palsy and their Physical, Social, and Attitudinal Environment: A Cross-Sectional Multicenter European Study*. Arch Phys Med Rehab* 2012**; 93**: 2154-2164.

27. Lindsay S. Child and Youth Experiences and Perspectives of Cerebral Palsy: A Qualitative Systematic Review*. Child Care Health Dev* 2016**; 42**: 153.

28. Thornton A, Licari M, Reid S, Armstrong J, Fallows R, Elliott C. Cognitive Orientation to (Daily) Occupational Performance Intervention Leads to Improvements in Impairments, Activity and Participation in Children with Developmental Coordination Disorder*. Disabil Rehabil* 2016**; 38**: 979-986.

29. Michelsen S, Flachs E, Uldall P, et al. Frequency of Participation of 8–12-Year-Old Children with Cerebral Palsy: A Multi-Centre Cross-Sectional European Study*. Eur J Paediatr Neurol* 2009**; 13**: 165-177.

30. Field D, Miller W, Jarus T, Ryan S, Roxborough L. Important Elements of Measuring Participation for Children Who Need Or use Power Mobility: A Modified Delphi Survey*. Dev Med Child Neurol* 2015**; 57**: 556-563.

31. Furtado SRC, Sampaio RF, Kirkwood RN, Vaz DV, Mancini MC. Moderating Effect of the Environment in the Relationship between Mobility and School Participation in Children and Adolescents with Cerebral Palsy*. Braz J Phys Ther* 2015**; 19**: 311-319.

32. Bedell G, Khetani M, Cousins M, Coster W, Law M. Parent Perspectives to Inform Development of Measures of Children's Participation and Environment*. Arch Phys Med Rehabil* 2011**; 92**: 765-773.

33. Kolehmainen N, Ramsay C, McKee L, Missiuna C, Owen C, Francis J. Participation in Physical Play and Leisure in Children with Motor Impairments: Mixed-Methods Study to Generate Evidence for Developing an Intervention*. Phys Ther* 2015**; 95**: 1374-1386.

34. Hwang A-, Yen C-, Liou T-, et al. Participation of Children with Disabilities in Taiwan: The Gap between Independence and Frequency*. PLoS ONE* 2015**; 10**.

35. Houtrow A, Newacheck P, Jones J, Ghandour R, Strickland B. Participation of Children with Special Health Care Needs in School and the Community*. Acad Pediatr* 2012**; 12**: 326-334.

36. Egilson ST, Traustadottir R. Participation of Students with Physical Disabilities in the School Environment*. Am J Occup Ther* 2009**; 63**: 264-272.

37. Peny-Dahlstrand M, Krumlinde-Sundholm L, Gosman-Hedstrom G. Patterns of Participation in School-Related Activities and Settings in Children with Spina Bifida*. Disabil Rehabil* 2013**; 35**: 1821-1827.

38. Raghavendra P, Olsson C, Sampson J, McInerney R, Connell T. School Participation and Social Networks of Children with Complex Communication Needs, Physical Disabilities, and Typically Developing Peers*. AAC Augmentative Altern Commun* 2012**; 28**: 33-43.

39. Wight M, Chapparo C. Social Competence and Learning Difficulties: Teacher Perceptions*. Aust Occup Ther J* 2008**; 55**: 256-265.

40. Webster AA, Carter M. Social Relationships and Friendships of Children with Developmental Disabilities: Implications for Inclusive Settings. A Systematic Review*. J Intellect Dev Disabil* 2007**; 32**: 200-213.

41. Parslow R, Beasant L, Johnson D, Crawley E, Patel A, Haywood K. What Matters to Children with CFS/ME? A Conceptual Model as the First Stage in Developing a PROM*. Arch Dis Child* 2015**; 100**: 1141-1147.

42. Sentenac M, Ehlinger V, Michelsen S, Marcelli M, Dickinson H, Arnaud C. Determinants of Inclusive Education of 8–12 Year-Old Children with Cerebral Palsy in 9 European Regions*. Res Dev Disabil* 2013**; 34**: 588-595.

43. Dolva A, Gustavsson A, Borell L, Hemmingsson H. Facilitating Peer Interaction--Support to Children with Down Syndrome in Mainstream Schools*. Eur J Spec Needs Educ* 2011**; 26**: 201-213.

44. Yalon-Chamovitz S, Mano T, Jarus T, Weinblatt N. Leisure Activities during School Break among Children with Learning Disabilities: Preference Vs. Performance*. Br J Learn Disabil* 2006**; 34**: 42-48.

45. Snyder PA, Rakap S, McLaughlin TW, Hemmeter ML, Sandall S, McLean ME. Naturalistic Instructional Approaches in Early Learning: A Systematic Review*. J Early Interv* 2015**; 37**: 69-97.

46. Eriksson L, Welander J, Granlund M. Participation in Everyday School Activities for Children with and without Disabilities*. J Dev Phys Disabil* 2007**; 19**: 485-502.

47. Almqvist L. Patterns of Engagement in Young Children with and without Developmental Delay*. J Policy Pract Intellect Disabil* 2006**; 3**: 65-75.

48. Rosenberg L, Ratzon NZ, Jarus T, Bart O. Perceived Environmental Restrictions for the Participation of Children with Mild Developmental Disabilities*. Child Care Health Dev* 2012**; 38**: 836-843.

49. Rosenberg L, Bart O, Ratzon NZ, Jarus T. Personal and Environmental Factors Predict Participation of Children With and Without Mild Developmental Disabilities*. J Child Fam Stud* 2013**; 22**: 658-671.

50. Pereira E, la Cour K, Jonsson H, Hemmingsson H. The participation experience of children with disabilities in Portuguese mainstream schools*. Br J Occup Ther* 2010**; 73**: 598-606.

51. Pivik J. The Perspective of Children and Youth: How Different Stakeholders Identify Architectural Barriers for Inclusion in Schools*. J Environ Psychol* 2010**; 30**: 510-517.

52. Barnett A, Dawes H, Wilmut K. Constraints and Facilitators to Participation in Physical Activity in Teenagers with Developmental Co-Ordination Disorder: An Exploratory Interview Study*. Child Care Health Dev* 2013**; 39**: 393-403.

53. Law MC, Darrah J, Pollock N, et al. Focus on function: a cluster, randomized controlled trial comparing child- versus context-focused intervention for young children with cerebral palsy*. Dev Med Child Neurol* 2011**; 53**: 621-629.

54. Coster W, Law M, Bedell G, et al. School Participation, Supports and Barriers of Students with and without Disabilities*. Child Care Health Dev* 2013**; 39**: 535-543.

55. Williams S, Leader G, Mannion A, Chen J. An Investigation of Anxiety in Children and Adolescents with Autism Spectrum Disorder*. Res Autism Spectr Disord* 2015**; 10**: 30-40.

56. Logan D, Simons L, Carpino E. Too Sick for School? Parent Influences on School Functioning among Children with Chronic Pain*. Pain* 2012**; 153**: 437-443.

57. Logan DE, Sieberg CB, Conroy C, Smith K, Odell S, Sethna N. Changes in Sleep Habits in Adolescents during Intensive Interdisciplinary Pediatric Pain Rehabilitation*. J Youth Adolesc* 2015**; 44**: 543-555.

58. Matsuoka M, Nagamitsu S, Iwasaki M, et al.  High Incidence of Sleep Problems in Children with Developmental Disorders: Results of a Questionnaire Survey in a Japanese Elementary School*. Brain Dev* 2014**; 36**: 35-44.

59. Carroll S, Moss-Morris R, Chalder T, Hemingway C, Heyman I. Understanding Fatigue in Paediatric Multiple Sclerosis: A Systematic Review of Clinical and Psychosocial Factors*. Dev Med Child Neurol* 2016**; 58**: 229-239.

60. Maher C, Watson A, Dollman J, et al. Fatigue is a Major Issue for Children and Adolescents with Physical Disabilities*. Dev Med Child Neurol* 2015**; 57**: 742-747.

61. Granlund M, Arvidsson P, Niia A, et al. Differentiating Activity and Participation of Children and Youth with Disability in Sweden: A Third Qualifier in the International Classification of Functioning, Disability, and Health for Children and Youth?*. Am J Phys Med Rehabil* 2012**; 91**: S84-S96.

62. Foster-Cohen S, Mirfin-Veitch B. Evidence for the Effectiveness of Visual Supports in Helping Children with Disabilities Access the Mainstream Primary School Curriculum*. J Res Spec Educ Needs* 2015.

63. Soref B, Ratzon NZ, Rosenberg L, Leitner Y, Jarus T, Bart O. Personal and Environmental Pathways to Participation in Young Children with and without Mild Motor Disabilities*. Child: Care Health Dev* 2012**; 38**: 561-571.

64. Colver AF, Dickinson HO, Parkinson K, et al. Access of Children with Cerebral Palsy to the Physical, Social and Attitudinal Environment they Need: A Cross-Sectional European Study*. Disabil Rehabil* 2011**; 33**: 28-35.

65. Berrin S, Malcarne V, Varni J, et al. Pain, Fatigue, and School Functioning in Children with Cerebral Palsy: A Path-Analytic Model*. J Pediatr Psychol* 2007**; 32**: 330-337.

66. Badia M, Riquelme I, Orgaz B, Acevedo R, Longo E, Montoya P. Pain, Motor Function and Health-Related Quality of Life in Children with Cerebral Palsy as Reported by their Physiotherapists*. BMC Pediatr* 2014**; 14**: 1.

67. Fauconnier J, Dickinson H, Beckung E, et al. Participation in Life Situations of 8-12 Year Old Children with Cerebral Palsy: Cross Sectional European Study*. Br Med J* 2009**; 338**: b1458.

68. Chen X, Gelaye B, Velez JC, et al. Attitudes, Beliefs, and Perceptions of Caregivers and Rehabilitation Providers about Disabled Children's Sleep Health: A Qualitative Study*. BMC Pediatr* 2014**; 14**: 245-245.

69. Parslow RM, Shaw A, Haywood KL, Crawley E. Important factors to consider when treating children with chronic fatigue syndrome/myalgic encephalomyelitis (CFS/ME): perspectives of health professionals from specialist services. *BMC Pediatrics* 2017**;17**: 43.

70. Piškur B, Beurskens AJ, Ketelaar M, Jongmans MJ, Casparie BM, Smeets RJ. Daily actions, challenges, and needs among Dutch parents while supporting the participation of their child with a physical disability at home, at school, and in the community: a qualitative diary study. *BMC pediatrics* 2017**; 17**: 12.

71. Øien I, Fallang B, Østensjø S. Everyday use of assistive technology devices in school settings. *Disability and Rehabilitation: Assistive Technology* 2016**; 11**: 630-635.

72. Ravenscroft J, Wazny K, Davis JM. Factors associated with successful transition among children with disabilities in eight European countries. *PloS one* 2017**; 12**: e0179904.

73. Chien CW, Rodger S, Copley J. Parent-reported Participation in Children with Moderate-to-severe Developmental Disabilities: Preliminary Analysis of Associated Factors using the ICF Framework. I*nternational Journal of Disability, Development and Education* 2017**; 64**: 483-496.

74. Chien CW, Branjerdporn G, Rodger S, Copley J. Exploring environmental restrictions on everyday life participation of children with developmental disability. *Journal of Intellectual & Developmental Disability* 2017**; 42**: 61-73.

75. Costa UM, Brauchle G, Kennedy-Behr A. Collaborative goal setting with and for children as part of therapeutic intervention. *Disability and rehabilitation* 2017**; 39**: 1589-1600.

76. Jindal P, MacDermid JC, Rosenbaum P, DiRezze B, Narayan A. Perspectives on rehabilitation of children with cerebral palsy: exploring a cross-cultural view of parents from India and Canada using the international classification of functioning, disability and health. *Disability and rehabilitation* 2018**; 40**: 2745-55.

77. Earde PT, Praipruk A, Rodpradit P, Seanjumla P. Facilitators and Barriers to Performing Activities and Participation in Children With Cerebral Palsy: Caregivers' Perspective. *Pediatric Physical Therapy* 2018**;30**: 27-32.

78. McGarty AM, Melville CA. Parental perceptions of facilitators and barriers to physical activity for children with intellectual disabilities: A mixed methods systematic review. *Research in developmental disabilities* 2018**; 73**: 40-57.

79. Bakanienė I, Žiukienė L, Vasiliauskienė V, Prasauskienė A. Participation of Children with Spina Bifida: A Scoping Review Using the International Classification of Functioning, Disability and Health for Children and Youth (ICF-CY) as a Reference Framework. *Medicina.* 2018**; 54** :40.

80. Anaby DR, Campbell WN, Missiuna C, Shaw SR, Bennett S, Khan S, Tremblay S, Kalubi‐Lukusa JC, Camden C, GOLDs (Group for Optimizing Leadership and Delivering Services). Recommended practices to organize and deliver school‐based services for children with disabilities: A scoping review. *Child: care, health and development* 2018.
